# Expression of *KNUCKLES* in the Stem Cell Domain Is Required for Its Function in the Control of Floral Meristem Activity in *Arabidopsis*

**DOI:** 10.3389/fpls.2021.704351

**Published:** 2021-07-21

**Authors:** Kamila Kwaśniewska, Caoilfhionn Breathnach, Christina Fitzsimons, Kevin Goslin, Bennett Thomson, Joseph Beegan, Andrea Finocchio, Nathanaël Prunet, Diarmuid S. Ó’Maoiléidigh, Frank Wellmer

**Affiliations:** ^1^Smurfit Institute of Genetics, Trinity College Dublin, Dublin, Ireland; ^2^Department of Molecular, Cell and Developmental Biology, University of California, Los Angeles, Los Angeles, CA, United States; ^3^Institute of Integrative Biology, University of Liverpool, Liverpool, United Kingdom

**Keywords:** stem cells, floral meristem, meristem termination, flower development, transcription factor

## Abstract

In the model plant *Arabidopsis thaliana*, the zinc-finger transcription factor KNUCKLES (KNU) plays an important role in the termination of floral meristem activity, a process that is crucial for preventing the overgrowth of flowers. The *KNU* gene is activated in floral meristems by the floral organ identity factor AGAMOUS (AG), and it has been shown that both AG and KNU act in floral meristem control by directly repressing the stem cell regulator *WUSCHEL* (*WUS*), which leads to a loss of stem cell activity. When we re-examined the expression pattern of *KNU* in floral meristems, we found that *KNU* is expressed throughout the center of floral meristems, which includes, but is considerably broader than the *WUS* expression domain. We therefore hypothesized that KNU may have additional functions in the control of floral meristem activity. To test this, we employed a gene perturbation approach and knocked down *KNU* activity at different times and in different domains of the floral meristem. In these experiments we found that early expression in the stem cell domain, which is characterized by the expression of the key meristem regulatory gene *CLAVATA3* (*CLV3*), is crucial for the establishment of *KNU* expression. The results of additional genetic and molecular analyses suggest that KNU represses floral meristem activity to a large extent by acting on *CLV3*. Thus, KNU might need to suppress the expression of several meristem regulators to terminate floral meristem activity efficiently.

## Introduction

The maintenance of stem cells in the shoot apical meristem is crucial for the growth and development of plants. Over the past 25 years, genetic and molecular studies have led to the identification of a number of key regulators of shoot meristem control and have begun to elucidate the mechanisms through which these regulators act. In the model plant *Arabidopsis thaliana*, genes involved in the CLAVATA–WUSCHEL pathway play a key role in the maintenance of shoot meristem activity and size by forming a regulatory feedback loop that connects the stem cell domain in the central zone of the shoot meristem with a group of underlying cells termed the organizing center (Kitagawa and Jackson, 2019). These genes include, among others, *CLAVATA1* (*CLV1*), *CLAVATA2* (*CLV2*), *CLAVATA3* (*CLV3*), and *WUSCHEL* (*WUS*). *CLV3*, which is expressed in the stem cells, encodes a small protein (Fletcher et al., 1999) that is proteolytically processed into a 12-amino acid peptide (Ito et al., 2006). This peptide is secreted from the stem cells and is thought to migrate to neighboring cells (Kitagawa and Jackson, 2019). *CLV1* is expressed in a domain underneath the stem cells and encodes a membrane-bound receptor kinase (Clark et al., 1997) that binds CLV3 peptides (Ogawa et al., 2008). CLV2 (Kayes and Clark, 1998) forms another CLV3 receptor together with the protein CORYNE (CRN) (Muller et al., 2008). Once activated, the receptors signal to repress *WUS* and restrict its expression to the cells of the organizing center underneath the stem cell domain (Brand et al., 2000; Schoof et al., 2000). A return signal from the organizing center to the stem cells is then provided directly by WUS, a homeodomain transcription factor (Mayer et al., 1998), which has been shown to move through plasmodesmata into the stem cell domain (Yadav et al., 2011; Daum et al., 2014) where it promotes *CLV3* expression (Brand et al., 2000; Schoof et al., 2000).

This central mechanism of shoot meristem control is also active in inflorescence and floral meristems (Kitagawa and Jackson, 2019), which are formed after the plant has switched from vegetative to reproductive growth. In contrast to shoot and inflorescence meristems, floral meristems are determinate structures that cease their activity once all floral organs have been initiated (Thomson and Wellmer, 2019). This termination process is crucial to prevent the overgrowth of flowers, a phenotype that can be seen in many mutants in which floral meristem termination is impaired. The regulatory mechanism for floral meristem termination is complex but much progress has been made in recent years to understand its structure and function (Xu et al., 2019). Key to this process is the floral organ identity factor AGAMOUS (AG), which controls the development of the reproductive floral organs, i.e., stamens and carpels (Yanofsky et al., 1990). *AG* expression commences at floral stage 3 in the center of floral meristems (Yanofsky et al., 1990). The AG transcription factor then activates genes that are specifically required for the formation of stamens and carpels (Gomez-Mena et al., 2005; ÓMaoiléidigh et al., 2013). At the same time, AG is also involved in the control of floral meristem activity. In fact, in loss-of-function *ag* mutants, floral meristems overgrow, leading to the formation of supernumerary floral whorls in the center of the flower (Yanofsky et al., 1990). It has been shown that AG mediates floral meristem control through both direct and indirect mechanisms. AG directly targets *WUS* and recruits Polycomb Group proteins to its promoter, leading to a gradual reduction in *WUS* expression through the deposition of repressive histone marks (Liu et al., 2011). AG also acts on *WUS* indirectly by activating the *KNUCKLES* (*KNU*) gene (Sun et al., 2009, 2014), which encodes a C2H2 zinc-finger transcription factor that is closely related to other important floral regulators such as SUPERMAN (SUP) (Payne et al., 2004). KNU contains an ERF-associated amphiphilic repression (EAR) motif at its carboxy terminus that has been shown to recruit transcriptional co-repressors and is thus thought to act primarily as a repressor of gene expression. *KNU* expression has been reported to commence at stage 6 and thus several stages after the onset of *AG* expression at stage 3 (Payne et al., 2004; Sun et al., 2009, 2019). The delay between AG activation and the expression of its target *KNU* appears to be due to a gradual removal of repressive marks from the *KNU* locus (Sun et al., 2014). Once KNU is expressed, it represses *WUS* and it has been suggested that this is mediated by direct binding of KNU to the *WUS* promoter (Sun et al., 2019). Two different, but not necessarily mutually exclusive mechanisms have been proposed for the repressive effect of KNU on *WUS*, which are both based on various epigenetic regulators being evicted from or recruited to the *WUS* locus (Bollier et al., 2018; Sun et al., 2019).

Despite the well-established role of KNU in floral stem cell termination, many questions about its function remain unanswered. For example, *knu* loss-of-function mutants exhibit phenotypes that are unlikely to be a result of impaired floral meristem control such as the formation of extended gynophores or male sterility defects (Payne et al., 2004). Thus, KNU has likely additional functions in flower development. Other questions relate to the control of the *KNU* expression pattern in floral meristems. While it is now well established, as outlined above, that AG activates *KNU*, how its spatial expression is restricted to the very center of the floral meristem in a domain that is much smaller than that of AG and later shifts to the basal end of developing gynoecia (Payne et al., 2004) is not understood.

In this study, we initially followed up on data we had obtained previously that suggested an earlier onset of *KNU* expression during flower development than what has been reported. To this end, we re-examined the expression pattern of *KNU* in floral meristems and found that *KNU* is indeed expressed slightly early than previously thought and in a domain that likely encompasses the entire 4th floral whorl. Notably this domain appears larger than the *WUS* expression domain and we therefore asked what other functions KNU may have in the control of floral meristem activity. Using a gene perturbation approach, in which we targeted *KNU* in different areas of the floral meristem, we found that the *CLV3*-expressing stem cell domain is crucial for KNU function. Results of additional genetic and molecular analyses suggest that KNU not only acts on *WUS* but also on *CLV3* to repress floral meristem activity. Thus, KNU may function as a general repressor of genes that control floral meristem activity.

## Materials and Methods

### Plant Materials and Growth

Plants were grown on a soil:vermiculite:perlite (3:1:1) mixture at 20–22°C under constant illumination. Published lines used for this study were as follows: pKNU::KNU-GUS (Sun et al., 2009); pKNU::KNU-VENUS (Sun et al., 2014); gAP3-GFP gSUP-3xVenusN7 pCLV3-dsRedN7 (Prunet et al., 2017); pAP1::AP1-GR *ap1-1 cal-1* (ÓMaoiléidigh et al., 2015); pCLV1::LhG4 (Schoof et al., 2000); pCLV3::LhG4 (Lenhard and Laux, 2003); pWUS::LhG4 (Gross-Hardt et al., 2002); *knu*-1(backcrossed into Landsberg *erecta*; Sun et al., 2009); *clv3-*1 (Clark et al., 1995); *crc-1* (Bowman and Smyth, 1999).

### Generation of Constructs and Transgenic Lines

For the construction of p35S::KNU-VP16, the *KNU* coding sequence was amplified with primers KK-180/KK-198 using cDNA derived from wild-type (L-*er*) inflorescences. The PCR product was then digested with *Eco*RI and ligated to a pBJ36-derived vector containing a p35S::VP16 cassette, which was also cleaved with *Eco*RI. The resulting p35S::KNU-VP16 cassette was released by *Not*I digestion and ligated to the *Not*I site of the binary vector pML-BART, which confers for ammonium-glufosinate resistance in plants. Colony PCR and genotyping assays for the detection of the construct were performed using primers KK-180/KK-35. To generate a version of p35S::KNU-VP16 with a mutated EAR motif (mEAR) the *KNU* coding sequence was amplified using primers KK-180/KK-196. This led to four leucine residues within the EAR motif being converted into alanines. The resulting PCR product was ligated into pML-BART (Eshed et al., 2001) as described above. The binary vectors were introduced into wild-type (L-*er*) plants using *Agrobacterium*-mediated transformation following the floral dip method (Clough and Bent, 1998). More than 25 independent transformants were analyzed per construct.

KNU-amiRNAs were designed using the Web microRNA WMD3 designer^1^ and constructed by overlapping PCR using the pRS300 plasmid (Schwab et al., 2006). Primers KK-31 to KK-34 were used for amiRNA1; primers KK-48 to KK-51 for amiRNA2; and primers KK-52 to KK-55 for amiRNA3. The resulting PCR products were cut with *Cla*I and *Xba*I and ligated into pBJ36 containing the 35S promoter or the 6xpOp promoter. After verifying the sequence of the amiRNA, a fragment containing the promoter and the amiRNA was sub-cloned into either the binary vectors pML-BART (p35S) or pART27 (Gleave, 1992) (6xpOp) using *Not*I sites. In order to generate lines for a dexamethasone-dependent induction of KNU-amiRNA expression, a pML-BART vector containing a p35S::GR-LhG4 cassette (Craft et al., 2005) was used together with the 6xpOp::KNU-amiRNA cassette described above. For the generation of p35S::KNU-amiRNA plants, the construct was transformed into wild-type (L-*er*) plants, and the following number of independent transformants was analyzed: 63 (amiRNA1); 82 (amiRNA2); 22 (amiRNA3). For the generation of p35S::GR-LhG4/6xpOp::KNU-amiRNA plants, the construct was transformed into wild-type (L-*er*) plants, resulting in the isolation of 72 independent transformants, which were characterized for phenotypes and *KNU* transcript levels after dexamethasone treatment.

For generating plants expressing the KNU-amiRNA1 in specific meristem domains, the vector pART27 6xpOp::KNU-amiRNA1 was transformed into the different LhG4 driver lines for *CLV1*, *CLV3* and *WUS*. First generation transformants (31 for pCLV1>>; 22 for pWUS>>; and 55 for pCLV3>>) were selected on MS plates containing kanamycin and genotyped with primers KK-35/KK-95.

To generate an epitope-tagged version of KNU for chromatin immunoprecipitation experiments, the primer combinations DM-90/DM-93 and DM-91/DM-92 were used to generate two PCR fragments that were subsequently used in an overlapping PCR with primers DM-90 and DM-91. Primers DM-92/DM-93 introduced synonymous nucleotide substitutions into the region of the *KNU* coding sequence that KNU-amiRNA1 targets (rKNU). The primers DM-90/91 introduced the restriction sites *Pst*I and *Eco*RI that were ∼2.6 kb upstream of the translational start site and at the end of the *KNU* coding sequence, respectively. This PCR product was then ligated to a pBJ36-derived vector containing the mGFP5 coding sequence followed by the 3′OCS terminator sequence. Once the presence of the KNU-encoding fragment was confirmed by colony PCR, this vector was cleaved using *Not*I and ligated to the binary vector pART27 that had been cleaved with *Not*I and treated with alkaline phosphatase (Roche). Plants containing the p35S::KNU-amiRNA1 transgene were transformed with this construct and lines in which the *knu*-like phenotypes of the amiRNA expressing line was rescued were isolated. First generation transformants were identified based on kanamycin resistance and were PCR genotyped using primers DM-59 and DM-31.

### GUS Staining

GUS staining was carried out as previously described^2^. In brief, flowers were harvested in 90% cold acetone and incubated for 15–20 min at room temperature and then vacuum infiltrated for 10 min. After removing the acetone, the staining solution containing X-gluc (C_1__4_H_1__3_BrClNO_7_) was added. The tissue was then again vacuum infiltrated until it sunk to the bottom of the tube, followed by incubation at 37°C. For sectioning, tissue was dehydrated through an ethanol series and treated with Formalin-Acetic-Alcohol fixative for 30 min. This was followed by incubation in 95% ethanol containing eosin. Tissue was later cleared with Tert-butanol (25 and 50% in ethanol) and incubated in 100% Tert-butanol overnight at 60°C. The following day, 50% of liquid paraplast was added and incubated for several hours at 60°C. The paraplast was then replaced a few times and ultimately placed into a weigh boat. Tissue in the hardened paraplast was cut into 8 μm sections using a Leica microtome and placed on slides that were incubated overnight at 42°C. Next, paraffin was removed by Histoclear (National Diagnostics) washes and staining was visualized using an Olympus SZX7 stereomicroscope.

### Imaging of Fluorescent Reporters

Shoot apices were prepared for imaging as previously described (Prunet et al., 2016, 2017; Prunet, 2017) and imaged using a Zeiss LSM880 confocal microscope using the Fast Airyscan mode.

### RNA Extraction and cDNA Synthesis

Total RNA was isolated from plant tissue using the Spectrum Plant Total RNA kit (Sigma-Aldrich) according to the manufacturer’s instructions. RNA was reverse transcribed using an oligo(dT)18 primer (Fermentas) and the RevertAid H Minus reverse transcriptase (Fermentas) according to the manufacturer’s instructions.

### Quantitative Reverse Transcription PCR (RT-qPCR)

For RT-qPCR, primers were designed to have a T_*m*_ of 60 ± 1°C. Each primer pair did not differ in annealing temperature of more than 1°C. In addition, primer pairs to amplify cDNA were designed to span exon-exon boundaries whenever possible and were as close to the 3‘end of the coding sequence as possible. A Lightcycler 480 (Roche) with a SYBR green master 1 (Roche) was used to quantify relative enrichments of cDNA. One reaction mix contained 5 μl of 2x SYBR green master 1, 1 μl cDNA, 1 μl of 10 μM primers and 3 μl of molecular biology grade water. An equivalent time of 60 s per 1 kb of DNA was given to generate the amplicon. Annealing temperatures between 58 and 60°C were used depending on the primer pairs. The reference gene used for RT-qPCR was At4g34270 (“REF2”), which was chosen from Czechowski et al. (2005). Primers used for RT-qPCR are listed in Supplementary Table 1. All RT-qPCR analyses were carried out with cDNA from at least 3 sets of biologically independent samples.

### Treatment of Plants With a Dexamethasone-Containing Solution and Tissue Collection

Once plants had bolted, the inflorescences of plants were treated with a solution containing 10 μM dexamethasone (Sigma) and 0.015% (v/v) Silwet-77 using a plastic Pasteur pipette. For the induction of amiRNA expression in the p35S::GR-LhG4/6xpOp::KNU-amiRNA lines, plants were treated trice with 3 days in-between treatments. For RT-qPCR analysis, tissue was collected from inflorescences of pAP1::AP1-GR *ap1-1 cal*-1 p35S::KNU-amiRNA1 plants as well as from pAP1::AP1-GR *ap1-1 cal-1* plants 5, 6, and 7 days after dexamethasone treatment.

### Chromatin Immunoprecipitation

For the analysis of KNU-GFP binding to the *CLV3* promoter region we collected inflorescence tissue from ∼4-weeks-old p35S::KNU-amiRNA pKNU::rKNU-mGFP5 pAP1::AP1-GR *ap1-1 cal-1* plants ∼7 days after dexamethasone treatment (as described above). Tissue was fixed in a solution of 1X PBS supplemented with 1% formaldehyde (Sigma 252549) at room temperature for 15 min under vacuum. Glycine was added to a final concentration of 0.125 M and a vacuum was applied for a further 5 min. The tissue was washed with dH_2_O three times. dH_2_0 was then removed and the tissue was frozen in LN_2_. The tissue was ground to fine powder using a pestle and mortar pre cooled with LN_2_ and incubated in 30 ml ice-cold extraction buffer 1 (Diagenode) supplemented with 0.1X protease inhibitors (Sigma P9599) for 5 min with gentle rocking at 4°C. The homogenate was filtered twice through Miracloth (Calbiochem) into precooled tubes and centrifuged at 3,000 × *g* for 20 min at 4°C. The pellet was suspended thoroughly in 1 ml ice-cold extraction buffer 2 (0.4 M sucrose, 100 mM Tris-HCl pH 8.0, 1% Triton X-100, 10 mM MgCl_2_, 0.1 mM phenylmethylsulfonyl fluoride, 5 mM β-mercaptoethanol, 0.1X protease inhibitors) and centrifuged at 12,000 × *g* for 10 min at 4°C. The pellet was washed and centrifuged once more with extraction buffer 2 and the supernatant was fully removed. The pellet was suspended in 300 μl ice-cold extraction buffer 3 (1.7 M sucrose, 100 mM Tris-HCl pH8.0, 0.15% (v/v) Triton X-100, 2 mM MgCl_2_, 0.1 mM phenylmethylsulfonyl fluoride, 5 mM β-mercaptoethanol, 0.1X protease inhibitors), transferred gently onto 300 μl extraction buffer 3 inside a 1.5 ml centrifuge tube, and centrifuged at 12,000 × *g* for 10 min at 4°C. The supernatant was removed and the pellet was suspended in 300 μl sonication buffer (Diagenode) and incubated on ice for 5 min. The nuclei suspension was sonicated using a Diagenode Bioruptor Pico for 7 cycles of 30 s on/off at 4°C, and centrifuged at 12,000 × *g* for 5 min at 4°C. The supernatant was frozen in LN_2_ and stored at –80°C until use.

Chromatin immunoprecipitation (ChIP) assays for each replicate were performed in triplicate and in parallel. Chromatin was thawed on ice and centrifuged at 12,000 × *g* for 5 min at 4°C to pellet insoluble material. The supernatant was diluted 5-fold in 1 X ChIP Dilution Buffer (CDB; Diagenode). Samples (200 μl diluted chromatin) were incubated with rotation at 4°C overnight with 6 μg of αGFP (Ab290; Abcam). 4 μl diluted chromatin was taken as input. 20 μl DiaMag protein A-coated magnetic beads were used per ChIP. The beads were washed 3 times in 200 μl CDB and suspended in their original volume before addition to the ChIP tube. Samples were incubated with the beads at 4°C with rotation for 2 h. The beads were washed once with each of wash buffers 1/2/3 (Diagenode) and twice with wash buffer 4 before suspension in 100 μl elution buffer 1 (Diagenode) and incubation with shaking at 65°C for 15 min. Four μl elution buffer 2 was added to each of the supernatants (separated from the beads) and incubated overnight at 65°C with shaking. ChIP DNA was purified from the eluates using the iPure kit v2 (Diagenode) and used for qPCR. ChIP-qPCR was performed using a Roche LightCycler 480 and SYBR Green I Master in a final volume of 10 μl. Primers used for qPCR analysis are listed in Supplementary Table 1.

### Image Processing

Some light microscopy images were processed to darken the background and to normalize the color to make the images clearer. These modifications do not alter the interpretation of the data, and all original unmodified images are available upon request.

## Results

### *KNU* Expression in Young Floral Buds

**FIGURE 1:**
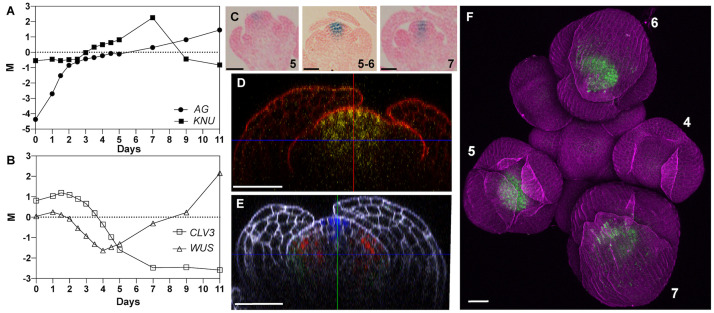
Expression of KNU in early stage floral buds. **(A,B)** Temporal expression of **(A)** *AG* and *KNU* and **(B)** *WUS* and *CLV3* during flower development. M values (log_2_ of expression of a gene at a given time-point/expression of that gene in a common reference sample) are shown. Data from Ryan et al. (2015). **(C)** Activity of a pKNU::KNU-GUS reporter in early stage flowers. Approximate floral stages are indicated. **(D)** Confocal image of a pKNU::KNU-VENUS reporter in a stage 5 floral bud. A vertical optical section is shown. **(E)** Confocal image of gAP3-GFP (green), gSUP-3xVenusN7 (red), and pCLV3-dsRedN7 (blue) reporters in a stage 5 floral bud. A vertical optical section is shown. Compare the expression domain of *CLV3* to that of *KNU* in **(D)**. **(F)** Confocal image of a pKNU::KNU-VENUS reporter in an inflorescence. Numbers indicate approximate floral stages. Scale bars: 20 μm.

*KNU* expression has been reported to commence at stage 6 of flower development in the stem cell domain as well as in cells of the underlying organizing center (Payne et al., 2004; Sun et al., 2009, 2019), which is crucial for meristem maintenance. When we analyzed data from an experiment in which we had monitored global gene expression during flower development from the time of initiation until maturation (Ryan et al., 2015), we found an onset of *KNU* up-regulation earlier than stage 6, around stage 4 or 5 (corresponding to days 3–4 in Figure 1A). For our previous study, we had used a floral induction system, which synchronizes flower development through the specific activation of the floral meristem identity regulator APETALA1 (AP1) in an *apetala1 cauliflower* (*ap1 cal*) double-mutant background (ÓMaoiléidigh et al., 2015). Because this system exhibits somewhat premature expression of the main *KNU* activator AG (Figure 1A), we considered the possibility that the observed early activation of *KNU* did not accurately reflect its expression in the wild type. To test this, we re-examined a published pKNU::KNU-GUS reporter line where the *KNU* coding region is translationally fused to β-glucuronidase (GUS) and driven by its native promoter (Sun et al., 2009). While the GUS activity pattern we observed in this experiment was very similar to what has been previously reported (Supplementary Figure 1), we detected GUS activity already at stage 5 in the center of floral meristems (Figure 1C). To independently verify this observation and to obtain detailed information on where in the floral meristem *KNU* is expressed, we imaged a published pKNU::KNU-VENUS reporter (Sun et al., 2014) using an Airyscan detector which provides improved resolution and signal-to-noise ratio relative to conventional confocal microscopy (Huff, 2015). This analysis confirmed an onset of *KNU* expression at stage 5 and showed expression in a domain that not only includes the stem cell domain and the underlying organizing center but most, if not all cells of the 4th floral whorl (Figures 1D–F). Thus, *KNU* expression in the floral meristem is broader and commences slightly earlier than previously thought.

### Effects of KNU-VP16 Expression on Plant Development

**FIGURE 2:**
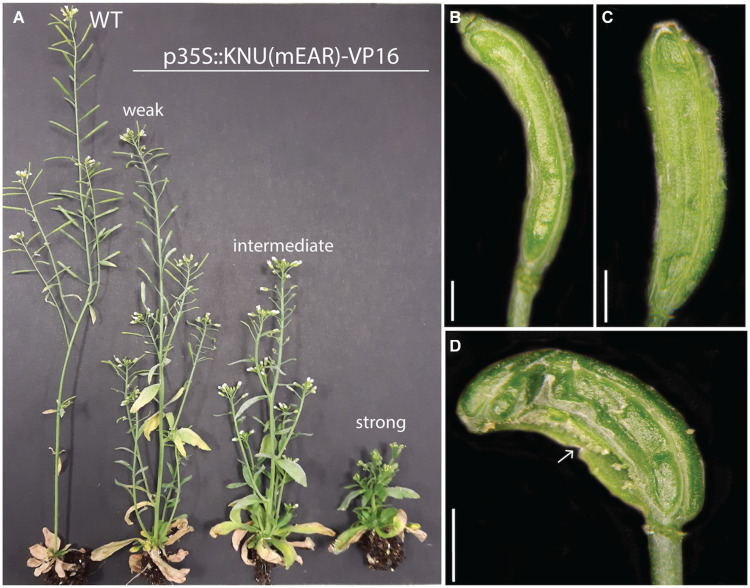
Effects of ectopic expression of a KNU(mEAR)-VP16 fusion protein. **(A)** A wild-type (WT) plant compared to plants carrying a p35S::KNU(mEAR)-VP16 transgene. Plants of different phenotypic strength are indicated. **(B)** Tricarpelloid silique of a phenotypically weak plant. **(C)** Tetracarpelloid silique of a phenotypically intermediate plant. **(D)** Silique of a phenotypically strong plant with fusion defects (arrow). Scale bars: 1 mm.

KNU is thought to terminate floral meristems by directly repressing the stem cell regulator *WUS* in the organizing center (Sun et al., 2009, 2019). Indeed, in the above-mentioned study where we monitored global gene expression during flower development, activation of *KNU* expression was followed by a reduction in the expression of *WUS* as well as of the stem cell marker *CLV3* (Figure 1B). While *CLV3* expression remained low at later stages of flower development, the expression of *WUS* increased again at intermediate stages likely as a consequence of its reported expression in stamens (Deyhle et al., 2007) and ovules (Gross-Hardt et al., 2002). As discussed above, the repressive activity of KNU is mediated by an EAR motif in its carboxy terminus. To test whether KNU acts exclusively as a repressor in floral meristem control, we expressed a fusion between KNU and the viral transcription activation domain VP16 (Triezenberg et al., 1988) from the constitutive Cauliflower Mosaic Virus (CaMV) 35S promoter in wild-type plants. It is known that the addition of VP16 can convert repressors into activators (Fujiwara et al., 2014) and in the case of KNU, expression of KNU-VP16 may therefore lead to overproliferation of floral meristems as observed in *knu* mutants. While plants carrying the p35S::KNU-VP16 construct showed some phenotypic abnormalities (Supplementary Figure 2), including floral defects such as shorter petals and siliques, these were not likely a consequence of altered meristem activity. We therefore considered the possibility that the presence of the EAR motif may prevent VP16 from being fully active and generated a version of the KNU-VP16 fusion protein in which four functionally important leucine residues in the EAR motif had been mutated to alanines (mEAR). Expression of this KNU(mEAR)-VP16 fusion protein from the 35S promoter in wild-type plants resulted in more severe defects than the expression of the fusion protein with an intact EAR domain. These transgenic plants were typically shorter in stature than the wild type (Figure 2A), likely as a consequence of the ectopic expression of the fusion protein, and exhibited a range of floral phenotypes (Supplementary Figure 3). These included the formation of enlarged gynoecia with supernumerary carpels (Figures 2B–D), suggesting that the conversion of the KNU repressor into an activator led to prolonged activity of the floral meristem. Thus, KNU may indeed act exclusively as a repressor in floral meristem control.

### Effects of Temporal *KNU* Perturbation

**FIGURE 3:**
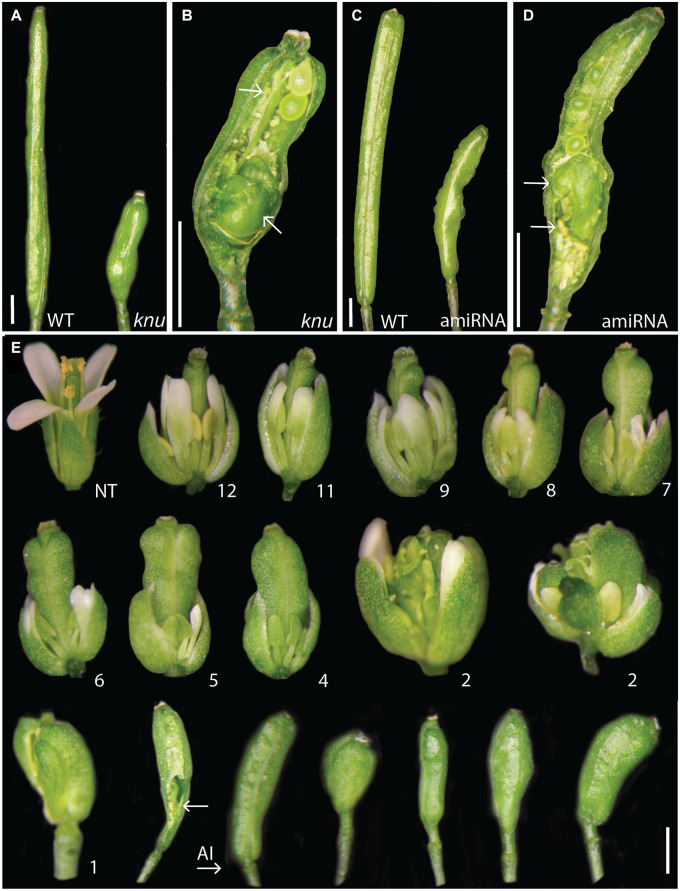
AmiRNA-mediated perturbation of *KNU* activity. **(A)** Siliques of a wild-type (WT) and a *knu*-1 plant. **(B)** Silique of a *knu-*1 plant with ectopic carpels and stamens (indicated by arrows). **(C)** Siliques of a wild-type plant and of a plant containing a p35S::KNU-amiRNA1 transgene. **(D)** Silique of a p35S::KNU-amiRNA1 plant with ectopic carpels and stamens (arrows). **(E)** Pulsed perturbation of *KNU*. Plants homozygous for a p35S::GR-LhG4/6xpOp::KNU-amiRNA1 transgene were treated three times (in 3-days intervals) with 10 μM dexamethasone. The effects of KNU-amiRNA induction were monitored for 3 weeks following the start of dexamethasone treatments by analyzing the phenotypes of flowers that reached maturity. Numbers indicate the approximate stage a flower was at the time of the first dexamethasone treatment. “NT”: a flower of an untreated plant resembling the wild type. Flowers shown on the right of “AI” likely initiated after the first dexamethasone treatment. In the bottom row, second flower from the left, an arrow marks ectopic organs in a *knu*-like silique (a valve has been partially removed to reveal internal structures). Size bars: 2 mm.

As described above, early *KNU* expression is found throughout most of the 4^th^ floral whorl. Its expression thus appears considerably broader than what would be required to repress *WUS* in the cells of the organizing center. We therefore asked whether KNU has additional functions in floral meristem control. To address this question, we sought to establish a gene perturbation approach and generated three artificial microRNAs (amiRNAs; Schwab et al., 2006) targeting *KNU*. When we expressed these amiRNAs from the constitutive 35S promoter in wild-type plants, we found two that led consistently to phenotypes resembling that of *knu* mutants, including shorter siliques, the formation of bulged siliques with ectopic organs, and extended gynophores (Figures 3A–D and Supplementary Figure 4). The penetrance of these floral phenotypes was overall reduced when compared to *knu* loss-of-function mutants likely due to an incomplete knockdown of *KNU* expression (see below). We used one of the functional amiRNAs (amiRNA1) to test when KNU function is required in the floral meristem. To this end, we generated transgenic plants that allow a specific expression of the KNU-amiRNA from the dexamethasone-inducible p35S::GR-LhG4/pOp promoter system (Craft et al., 2005). We then activated KNU-amiRNA expression by treating inflorescences three times (with 3-day intervals) with dexamethasone and subsequently monitored, over a 3-weeks period, phenotypic effects in flowers that reached maturity. In this experiment, *knu*-like phenotypes were observed primarily in flowers that were at very early stages (<stage 4) at the time of the first dexamethasone treatment or initiated after the onset of amiRNA expression (Figure 3E). Thus, the KNU-amiRNAs that accumulated in very young floral buds likely interfered with the onset of *KNU* expression at stage 5, hence supporting the idea that the earliest expression of *KNU* is crucial for floral meristem control.

**FIGURE 4:**
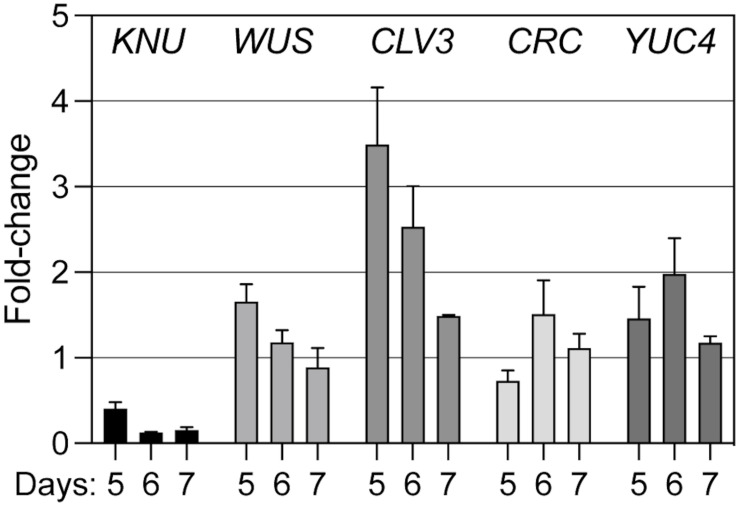
Effect of amiRNA-mediated perturbation of *KNU* on the expression of meristem regulators. A p35S::KNU-amiRNA transgene was introgressed into pAP1::AP1-GR *ap1-1 cal-1* plants. Flower development in this line, as well as in pAP1::AP1-GR *ap1-1 cal-1* plants that did not express the KNU-amiRNA, was induced by dexamethasone treatment and floral buds were collected 5, 6, and 7 days after the treatment. Transcript levels of different meristem regulators (as indicated) were determined by RT-qPCR in four (5 and 6 days) or three (7 days) biologically independent sets of samples. Fold change expression values in the amiRNA-expressing lines relative to the lines with normal KNU activity are shown. Bars indicate SEM.

**FIGURE 5:**
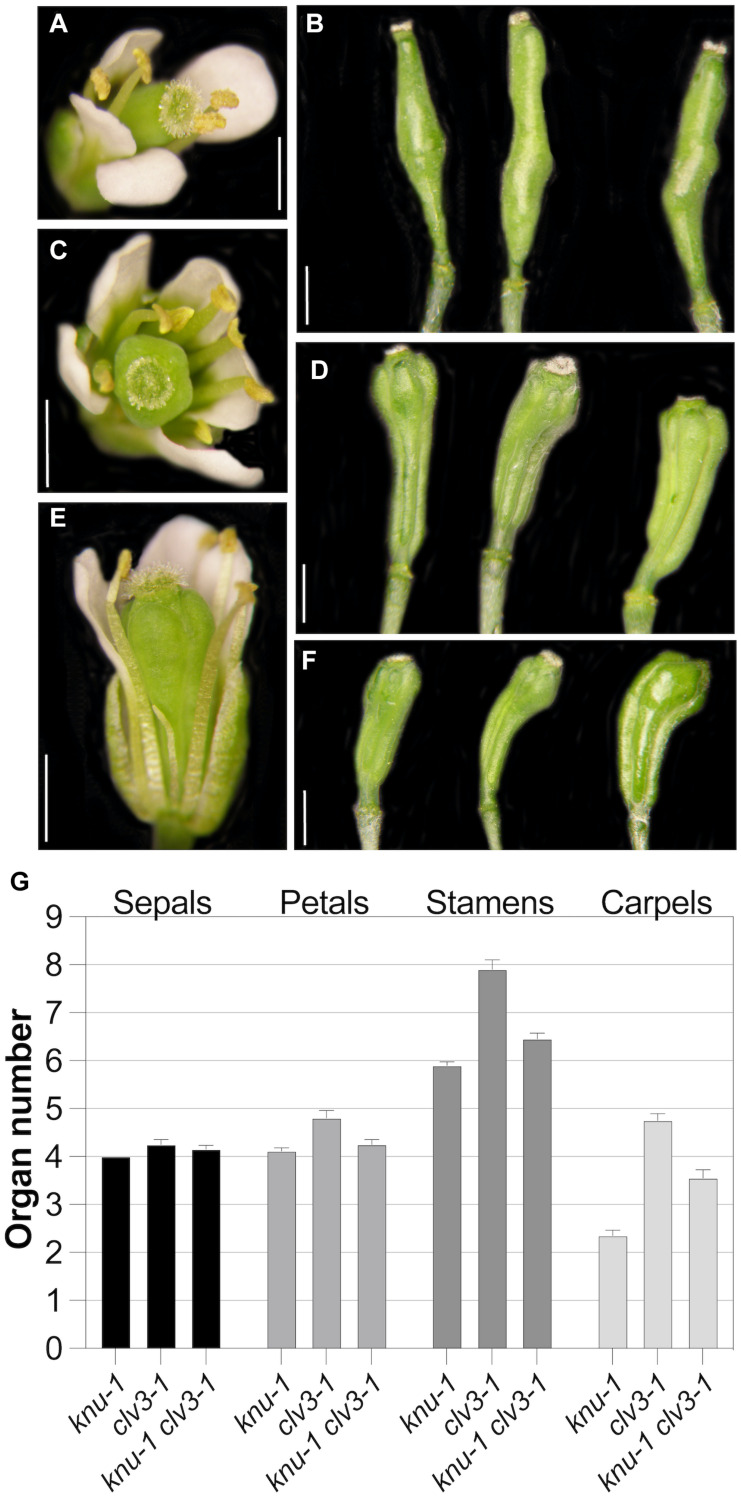
Genetic interaction between *knu-1* and *clv3-1*. **(A,B)** Floral phenotypes of *knu-1* in an L-*er* background. A flower **(A)** and bulged siliques **(B)** are shown. **(C,D)** Floral phenotypes of *clv3-1* mutant plants. A flower with an increased number of floral organs **(C)** and multicarpelled siliques **(D)** are shown. **(E,F)** Floral phenotypes of *knu-1 clv3-1* double-mutant plants. A flower **(E)** and siliques **(F)** are shown. In **(E)**, a petal was removed for better visibility of internal organs. Siliques shown in **(F)** were shorter than those of *clv3-1* single mutants **(D)**. **(G)** Number of floral organs in *knu-1*, c*lv3-1* and *knu-1 clv3-1* flowers. *n* = 20. Error bars indicate SEM. Size bars: 1 mm.

We next tested the effects of *KNU* perturbation on the expression of selected regulators of floral meristem activity. To this end, we crossed a p35S::KNU-amiRNA transgene into the background of the above-mentioned floral induction system and induced flower development in the resulting line as well as in plants of the floral induction system that did not contain the p35S::KNU-amiRNA transgene. We then collected floral buds 5, 6, and 7 days after the induction, which correspond approximately to floral stages 6–7 (5 days), 7–8 (6 days), and 8 (7 days), respectively (Ryan et al., 2015), thus including the stages immediately following floral meristem termination in the wild type. Using quantitative reverse transcription PCR (RT-qPCR) we then measured transcript levels of selected floral meristem regulators (Figure 4). As expected, the expression of *KNU* was significantly (*p* < 0.01; ratio paired *t*-test) decreased at all time-points in plants expressing the KNU-amiRNA but this knockdown was incomplete, likely explaining the weaker phenotypes of p35S::KNU-amiRNA lines when compared to *knu* loss-of-function mutants (see above). Expression of *WUS* was significantly (*p* < 0.05; ratio paired *t*-test) increased at the 5-days time-point but not at later time-points. In contrast, expression of the stem cell marker *CLV3* was markedly upregulated in plants expressing the KNU-amiRNA at the 5- and 6-days time-points (*p* < 0.01 and *p* < 0.05, respectively, ratio paired *t*-test). Because it has been shown that *KNU* genetically interacts with *CRABS CLAW* (*CRC*) (Yamaguchi et al., 2017); see also Supplementary Figure 5), a transcription factor-coding gene that has been implicated in floral meristem control (Yamaguchi et al., 2017), we included it, as well as its target *YUCCA4* (*YUC4*) (Yamaguchi et al., 2018), in this analysis. The effect of *KNU* perturbation on the expression of these genes appeared limited with the exception of *YUC4* in the 6-d time point. Thus, from the floral meristem regulators tested in this experiment, the strongest effect of a *KNU* knockdown was observed for *CLV3*. Because *KNU* is expressed in the *CLV3*-expressing stem cell domain (see above), we asked whether the observed increase in *CLV3* transcript levels after *KNU* perturbation may be due to KNU repressing *CLV3*, either by directly binding to its promoter, or indirectly, e.g., via mis-regulation of *WUS*. To test this, we first generated a double mutant between *knu-1* (Payne et al., 2004; Sun et al., 2009) and the intermediate *CLV3* allele *clv3-1* (Clark et al., 1995), which carries a mutation in the coding region of the gene, leading to a Gly75Arg substitution (Fletcher et al., 1999). We reasoned that if KNU was indeed repressing *CLV3* then removal of *KNU* activity may partially restore *CLV3* activity. In agreement with this idea we found that *knu-1 clv3-1* double mutants exhibited a reduction (*p* < 0.0001; *t*-test) in the number of stamens and carpels relative to *clv3-1* single-mutant plants (Figure 5). To test whether KNU targets *CLV3* directly, we generated a pKNU::KNU-GFP line in the background of the above-mentioned floral induction system and carried out chromatin immunoprecipitation experiments using a GFP antiserum coupled to quantitative PCR. While the results of these experiments suggest enrichment of certain *CLV3* promoter regions (Supplementary Figure 6), the data obtained in independent replicate experiments were variable likely due to low levels of KNU protein and/or its spatially restricted domain of expression. Therefore, experimental approaches with increased sensitivity may be required to show unequivocally that KNU binds to the *CLV3* locus (see section “Discussion”).

### Effects of Spatial *KNU* Perturbation

**FIGURE 6:**
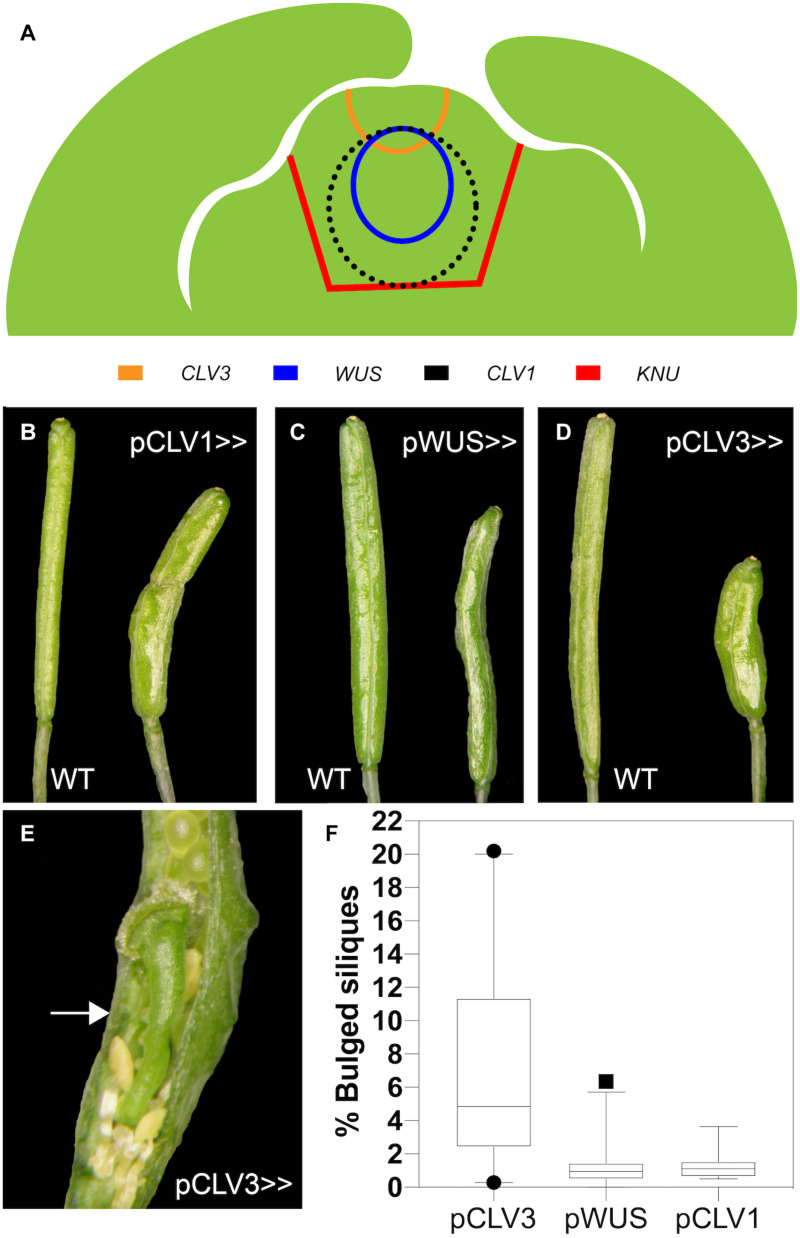
Effects of expression of the KNU-amiRNA from different driver lines. **(A)** Schematic of a cross-section of a stage 5 floral bud. The approximate expression domains of *KNU*, *CLV1*, *CLV3*, and *WUS* are indicated. **(B–D)** Siliques of the wild-type (on the left) and of driver lines (on the right) for *CLV1* **(B)**, *WUS* **(C)** and *CLV3* **(D)** expressing the KNU-amiRNA1. **(E)** Silique of a plant expressing the KNU-amiRNA from the *CLV3* driver. An arrow marks an ectopic gynoecium. Ectopic stamens are also visible. Part of a valve has been removed to reveal the internal structures. **(F)** Quantification of phenotypic effects. The percentage of siliques with bulges was determined in lines expressing the KNU-amiRNA from the different driver lines (as indicated). Black dots and a square indicate outliers. Data are based on the analysis of at least 16 independent transformants per driver line.

The results discussed above suggest that KNU function in the control of floral meristem determinacy may not be restricted to the organizing center and the control of *WUS* expression. To further test this idea, we sought to perturb *KNU* activity in different domains of the floral meristem (Figure 6A) and to assess the resulting effects on meristem determinacy. To this end, we employed previously established and characterized driver lines for *CLV1*, *CLV3*, or *WUS* (Schoof et al., 2000; Gross-Hardt et al., 2002; Lenhard and Laux, 2003). In these lines, the synthetic transcription factor LhG4 (Moore et al., 1998) is expressed from the promoters of the different meristem regulators, leading to the domain-specific activation of a transcript of interest that is under control of the pOp promoter which is targeted by LhG4. We first assessed the strength of these driver lines so that we could relate their activities to the results from subsequent *KNU* perturbation experiments. To this end, we introduced a 6xpOp::GUS transgene into the different driver lines and carried out staining reactions for 1 and 2 h. After 1 h, we detected GUS activity in early-stage floral buds of the *WUS* driver line but not in the driver lines for *CLV1* and *CLV3* (Supplementary Figure 7). After 2 h, staining was detected in inflorescences of both the *WUS* and *CLV3* driver lines but not readily in lines containing the *CLV1* promoter. However, a closer examination of the *CLV1* driver line through sectioning showed GUS signal in a domain underneath the stem cells after 2 h of staining (Supplementary Figure 7), indicating that the driver was indeed active. Thus, of the three driver lines used, the *WUS* driver appeared to be strongest, followed by the *CLV3* and the *CLV1* drivers.

**FIGURE 7:**
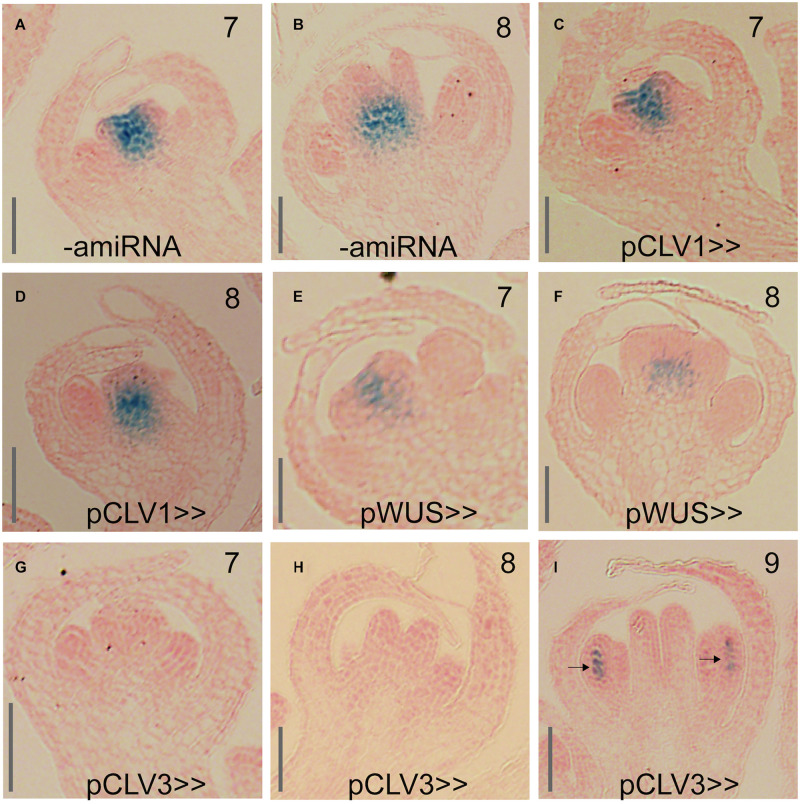
Effect of KNU-amiRNA expression from different driver lines on *KNU* expression. **(A,B)** pKNU::KNU-GUS activity in plants that do not express the KNU-amiRNA. **(C,D)** pKNU::KNU-GUS activity in plants expressing the KNU-amiRNA from the *CLV1* driver. **(E,F)** pKNU::KNU-GUS activity in plants expressing the KNU-amiRNA from the *WUS* driver. **(G–I)** pKNU::KNU-GUS activity in plants expressing the KNU-amiRNA from the *CLV3* driver. In **(I)**, GUS signal in anthers is marked by arrows. Approximate floral stages are indicated by numbers. Scale bars: 25 μm.

To perturb *KNU* in different domains of the floral meristem, we next introduced a pOp::KNU-amiRNA transgene into the driver lines (referred to hereafter as pCLV1>>KNU-amiRNA, pCLV3>>KNU-amiRNA and pWUS>>KNU-amiRNA) and assessed independent transformants for floral phenotypes. Notably, the *CLV1*, *CLV3* and *WUS* are already active in stage 2 floral buds (Clark et al., 1997; Mayer et al., 1998; Fletcher et al., 1999) so that cells within their expression domains should accumulate KNU-amiRNAs before *KNU* expression commences at stage 5, which may be important for the efficient removal of *KNU* transcripts (see above). Because the *WUS* driver line appeared stronger than the driver lines for the *CLV* genes and repression of *WUS* by KNU is thought to be essential for floral meristem control, we expected that a perturbation of *KNU* in the *WUS* domain would result in the strongest phenotypic effects. At the same time, due to the CLV-WUS feedback loop, an upregulation of *WUS* as a result of *KNU* perturbation would likely lead to increased *CLV3* levels in the stem cells, which may negatively affect the activity of the *WUS* driver. When we analyzed independent transformants carrying the pWUS>>KNU-amiRNA construct we found that they showed only minor defects with siliques being sometimes shorter than those of the wild type but only rarely containing ectopic organs (Figures 6C,F). Similar results were obtained for the *CLV1* driver lines (Figures 6B,F). In contrast, expression of the KNU-amiRNA from the *CLV3* driver resulted in much stronger phenotypes with the frequent formation of bulged siliques that contained ectopic organs (Figures 6D–F), thus resembling siliques of *knu* mutants. We next investigated whether the different phenotypes were a result of differences in the degree of *KNU* perturbation. To this end, we crossed the above-mentioned pKNU::KNU-GUS reporter into the lines expressing the KNU-amiRNA under control of the different drivers. *KNU* expression was largely unaffected in pCLV1>>KNU-amiRNA lines relative to plants that did not express the amiRNA (Figures 7A–D) possibly as a result of the comparatively weak activity of the *CLV1* driver. KNU-GUS activity in the pWUS>>KNU-amiRNA lines appeared reduced but was still readily detectable (Figures 7E,F). In contrast, in the pCLV3>>KNU-amiRNA lines, *KNU* expression was completely absent from early stage flowers (Figures 7G,H). At later floral stages, *GUS* activity was detected in anthers (Figure 7I) as in the wild type (Supplementary Figure 1), presumably because the *CLV3* driver is not active in stamens (Lenhard and Laux, 2003). Thus, the degree of *KNU* down-regulation in the different driver lines matched the strength of the phenotypic effects observed. Taken together, the results from these experiments show that *KNU*-amiRNA expression in the *CLV3* domain efficiently removes *KNU* expression from the floral meristem, suggesting that the stem cell domain is essential for KNU function in floral meristem control.

## Discussion

In this study, we analyzed the function of the KNU transcription factor, which is an important regulator of floral meristem activity. *KNU* expression has been previously reported to commence at stage 6 in the center of floral meristems and to be confined mainly to the organizing center with weaker expression in the outermost stem cell layers (Sun et al., 2009). The same group later concluded that early *KNU* expression is confined mainly to the central zone and then spreads to cells of the organizing center, resulting in a transient overlap between the expression domains of *KNU* and *WUS* in stage 6 flowers (Sun et al., 2019). Because in a previous study (Ryan et al., 2015) we had obtained data that suggested an onset of *KNU* expression earlier than stage 6, we re-examined its expression pattern using the reporter lines established by Sun and colleagues. This analysis showed an onset of *KNU* expression at stage 5 (Figure 1) and thus slightly earlier than previously thought. What’s more, the early expression domain of *KNU* appeared to be extending beyond the stem cell domain and the organizing center and to cover most, if not all, of the 4th floral whorl (Figure 1).

Because *KNU* expression seems broader than what would be needed to repress *WUS* in the cells of the organizing center, we asked whether KNU may have other functions in the floral meristem and where in the floral meristem KNU activity is needed for stem cell termination. To test this, we employed a gene perturbation approach and expressed a functional KNU-amiRNA from previously established and characterized driver lines for *CLV1*, *CLV3* and *WUS*. We then assessed the floral phenotypes in the resulting transgenic lines (Figure 6) and determined effects on *KNU* expression using a pKNU::KNU-GUS reporter (Figure 7). In these experiments, we found that expression of the KNU-amiRNA from the *CLV1* driver had little effect on either floral meristem determinacy or *KNU* expression likely as a result of the comparatively low activity of the driver used. Expression of the KNU-amiRNA from the *WUS* driver also resulted in only weak effects on floral meristem activity although *KNU* expression appeared to be somewhat reduced in these lines. This result was surprising, given that the suppression of *WU*S by KNU in the cells of the organizing center is thought to be essential for floral meristem termination. One possible explanation for the limited effect observed in this experiment is the fact that an upregulation of *WUS* as a result of the amiRNA-dependent perturbation of *KNU* is likely to lead to increased *CLV3* levels in the stem cell domain, which may then negatively affect the activity of the *WUS* driver leading to a reduction in amiRNA expression. Alternatively, residual KNU activity in these lines may have been sufficient for *WUS* repression. In contrast to the *CLV1* and *WUS* driver lines, expression of the KNU-amiRNA from the *CLV3* driver led to *knu* mutant-like phenotypes and a strong reduction of *KNU* expression throughout the center of floral meristems.

It has been demonstrated that small RNAs can be mobile within plants and travel in-between cells (Molnar et al., 2010). We therefore considered the possibility that the strong effect of KNU-amiRNA expression from the *CLV3* driver may be a result of movement of the amiRNA from the central zone into adjacent cell layers. However, this seems highly unlikely as it has been demonstrated that miRNAs act autonomously within the *CLV3* and *WUS* expression domains, suggesting that the movement of small RNAs is either completely blocked or largely restricted within the stem cell niche (Skopelitis et al., 2018). We also considered whether the results of the perturbation experiments could be a consequence of differences in the strength of the driver lines used, which may have led to different knockdown efficiencies. While we did indeed detect some differences in the strength of these lines (Supplementary Figure 7), at least in the case of *CLV3* and *WUS* these did not mirror the strength of the phenotypic effects observed with the different driver lines. We therefore concluded that the *CLV3*-expressing stem cell domain is essential for KNU function either because it is needed for the onset, establishment and maintenance of *KNU* expression and/or because KNU acts on additional target genes in this region. A strong candidate for such a target was *CLV3* itself. We therefore tested whether *CLV3* is differentially expressed in early stage flowers in which *KNU* function had been perturbed by the expression of the KNU-amiRNA. In this experiment, we found that *CLV3* transcript levels were increased and that the expression differences we detected were higher for *CLV3* than those for *WUS* (Figure 4). This result is in agreement with data from Sun and colleagues, who showed that the specific activation of KNU in flowers leads to a rapid down-regulation of *CLV3* transcript levels (Sun et al., 2019), suggesting that this may be a direct effect. In agreement with the idea of KNU repressing *CLV3*, we found that the phenotype of the intermediate *CLV3* allele *clv3-1* was partially rescued by introgression of a *knu* loss-of-function allele (Figure 5). However, this interaction could be indirectly mediated by *WUS*, which is upregulated in *knu* mutants, possibly leading to increased *CLV3* expression. We therefore tested whether KNU represses *CLV3* directly and carried out chromatin immunoprecipitation experiments. While we did obtain data in support of a direct regulation (Supplementary Figure 6), a high degree of variability in these experiments did not allow us to conclude unequivocally that this regulation is indeed direct. Thus, approaches with increased sensitivity, such as CUT and RUN (Skene and Henikoff, 2017), may be required in the future to identify KNU target genes with high confidence and on a genome-wide scale. Taken together our results suggest that KNU is mediating floral meristem termination through the direct or indirect repression of not only *WUS* but also of *CLV3*, and given the broader than thought expression domain of *KNU* in the center of floral meristems, perhaps of additional meristem regulators.

## Data Availability Statement

The original contributions presented in the study are included in the article/Supplementary Material, further inquiries can be directed to the corresponding author/s.

## Author Contributions

KK, DÓ’M, and FW conceived and planned the experiments. FW supervised the research and wrote the manuscript. All authors carried out the experiments, analyzed the data, read the manuscript, and made comments for improvements.

## Conflict of Interest

The authors declare that the research was conducted in the absence of any commercial or financial relationships that could be construed as a potential conflict of interest.

## Footnotes

1 wmd3.weigelworld.org

2 www.arabidopsis.org/download_files/Protocols/EMBOmanual/ch9.pdf

## Supplementary Material

Supplementary Figure 1 Activity of a pKNU::KNU-GUS reporter across flower development. **(A,B)** GUS activity in the center of a stage 6 **(A)** and 7 **(B)** flower. **(C–E)** Later in development, GUS activity was observed at the base of the gynoecium. **(E)** At stage 9, GUS activity was also present in the anthers of stamens. **(F–I)** At later stages (∼10–13), activity of the pKNU::KNU-GUS reporter decreased. **(H)** GUS activity detected at the base of a developing gynoecium of a stage 11 flower. **(I)** GUS activity in the stigmatic tissue of mature flowers. Scale bars: 50 μm.

Supplementary Figure 2 Phenotypes of p35S::KNU-VP16 plants. **(A)** Flower of a transgenic plant with reduced size compared to the wild type, and numerous trichomes on sepals. **(B)** Size comparison of a wild-type (WT) petal and a petal of a transgenic plant. **(C)** Plants carrying the p35S::KNU-VP16 transgene produced smaller leaves than the wild type. **(D)** Siliques of wild-type and p35S::KNU-VP16 plants. **(E)** Silique of a transgenic plant with reduced seed set. Part of a valve was removed for better visibility. Scale bars: 1 mm.

Supplementary Figure 3 Floral phenotypes of p35S::KNU(mEAR)-VP16 plants. **(A)** Flower of a phenotypically weak line with a bent gynoecium. **(B–D)** Flowers of lines with intermediate phenotypes with mild homeotic transformations. Arrows indicate a stamen with carpelloid tissue **(B)**; a petalloid stamen **(C)**; and a sepalloid petal **(D)**. **(E)** Flower from a phenotypically strong plant with misshapen and green petals. The tricarpelloid gynoecium exhibited reduced seed set. **(F)** Flower with sepal-like organs in place of petals and stamens and an unfused gynoecium. Sepals were removed from all flowers for better visibility of the inner-whorl organs.

Supplementary Figure 4 Identification of functional *KNU*-amiRNAs. **(A)** Schematic drawing showing the sequences of the three amiRNAs tested in this study and the regions in *KNU* they are targeting. **(B)** Percentage of bulged siliques formed by lines expressing the different amiRNAs (as indicated). At least 20 independent transformants were analyzed per construct. **(C,D)** Siliques of p35S::KNU-amiRNA2 plants were bulged and often contained ectopic carpels and stamens (indicated by arrows). **(E,F)** Siliques of p35S::KNU-amiRNA3 plants. No ectopic organ formation was observed **(F)**, however, siliques were often tricarpelloid (arrow) **(E)**.

Supplementary Figure 5 Genetic interaction between *CRC* and *KNU* in the control of floral meristem activity. **(A)** *Crc-1* mutant flower with unfused carpel at the top of the gynoecium. **(B)** *knu-1 crc-1* double-mutant flower with shorter stamens, which do not produce pollen, and ectopic tissue inside the gynoecium. **(C)** Early arising silique of the double mutant with an unfused gynoecium and ectopic carpel-like tissue. **(D)** Silique from a late arising flower producing a new flower from a silique. **(E)** Siliques of p35S::KNU-amiRNA1 *crc-1* plants were similar to siliques of *knu-1 crc-1* double mutants - compare to **(C)**.

Supplementary Figure 6 Results of chromatin immunoprecipitation experiments. ChIP-qPCR analysis of KNU-GFP binding to the *CLV3* promoter region in ∼stage 7-8 floral buds.**(A)** Position of primer pairs used to analyze KNU-GFP binding across the *CLV3* promoter region. **(B)** ChIP analysis of KNU-GFP binding to the *CLV3* promoter locus. Data is shown as fold enrichment of target loci over enrichment of reference genes ACT, MU and TUB (see section “Materials and Methods”). Three independent experiments were performed and the mean value is plotted above with error bars denoting SEM.

Supplementary Figure 7 Characterization of the strength of the driver lines used. **(A,B)** Inflorescences of a pWUS>>GUS line after staining for 1 **(A)** or 2 **(B)** hours. **(C,D)** Inflorescences of a pCLV3>>GUS line after staining for 1 **(C)** or 2 **(D)** hours. **(E,F)** Inflorescences of a pCLV1>>GUS line after staining for 1 **(E)** or 2 **(F)** hours. **(G,H)** Inflorescences of a pCLV1>>GUS line were sectioned after staining for 1 **(G)** or 2 **(H)** hours. Scale bars: 20 μm.

Supplementary Table 1 List of primers used in this study.

## References

[B1] Bollier N. Sicard A. Leblond J. Latrasse D. Gonzalez N. Gevaudant F. (2018). At-MINI ZINC FINGER2 and Sl-INHIBITOR OF MERISTEM ACTIVITY, a conserved missing link in the regulation of floral meristem termination in *Arabidopsis* and tomato. *Plant Cell* 30 83–100. 10.1105/tpc.17.00653 29298836 PMC5810569

[B2] Bowman J. L. Smyth D. R. (1999). CRABS CLAW, a gene that regulates carpel and nectary development in *Arabidopsis*, encodes a novel protein with zinc finger and helix-loop-helix domains. *Development* 126 2387–2396. 10.1242/dev.126.11.2387 10225998

[B3] Brand U. Fletcher J. C. Hobe M. Meyerowitz E. M. Simon R. (2000). Dependence of stem cell fate in *Arabidopsis* on a feedback loop regulated by CLV3 activity. *Science* 289 617–619. 10.1126/science.289.5479.617 10915624

[B4] Clark S. E. Running M. P. Meyerowitz E. M. (1995). CLAVATA3 is a specific regulator of shoot and floral meristem development affecting the same processes as CLAVATA1. *Development* 121 2057–2067. 10.1242/dev.121.7.2057

[B5] Clark S. E. Williams R. W. Meyerowitz E. M. (1997). The CLAVATA1 gene encodes a putative receptor kinase that controls shoot and floral meristem size in *Arabidopsis*. *Cell* 89 575–585. 10.1016/s0092-8674(00)80239-1 9160749

[B6] Clough S. J. Bent A. F. (1998). Floral dip: a simplified method for Agrobacterium-mediated transformation of *Arabidopsis thaliana*. *Plant J.* 16 735–743. 10.1046/j.1365-313x.1998.00343.x 10069079

[B7] Craft J. Samalova M. Baroux C. Townley H. Martinez A. Jepson I. (2005). New pOp/LhG4 vectors for stringent glucocorticoid-dependent transgene expression in *Arabidopsis*. *Plant J.* 41 899–918. 10.1111/j.1365-313x.2005.02342.x 15743453

[B8] Czechowski T. Stitt M. Altmann T. Udvardi M. K. Scheible W. R. (2005). Genome-wide identification and testing of superior reference genes for transcript normalization in *Arabidopsis*. *Plant Physiol.* 139 5–17. 10.1104/pp.105.063743 16166256 PMC1203353

[B9] Daum G. Medzihradszky A. Suzaki T. Lohmann J. U. (2014). A mechanistic framework for noncell autonomous stem cell induction in *Arabidopsis*. *Proc. Natl. Acad. Sci. U.S.A.* 111 14619–14624. 10.1073/pnas.1406446111 25246576 PMC4210042

[B10] Deyhle F. Sarkar A. K. Tucker E. J. Laux T. (2007). WUSCHEL regulates cell differentiation during anther development. *Dev. Biol.* 302 154–159. 10.1016/j.ydbio.2006.09.013 17027956

[B11] Eshed Y. Baum S. F. Perea J. V. Bowman J. L. (2001). Establishment of polarity in lateral organs of plants. *Curr. Biol.* 11 1251–1260. 10.1016/s0960-9822(01)00392-x 11525739

[B12] Fletcher J. C. Brand U. Running M. P. Simon R. Meyerowitz E. M. (1999). Signaling of cell fate decisions by CLAVATA3 in *Arabidopsis* shoot meristems. *Science* 283 1911–1914. 10.1126/science.283.5409.1911 10082464

[B13] Fujiwara S. Kigoshi K. Mitsuda N. Suzuki K. Ohme-Takagi M. (2014). VP16 fusion efficiently reveals the function of transcriptional repressors in *Arabidopsis*. *Plant Biotechnol.* 31 123–132. 10.5511/plantbiotechnology.14.0121a

[B14] Gleave A. P. (1992). A versatile binary vector system with a T-DNA organisational structure conducive to efficient integration of cloned DNA into the plant genome. *Plant Mol. Biol.* 20 1203–1207. 10.1007/bf00028910 1463857

[B15] Gomez-Mena C. De Folter S. Costa M. M. Angenent G. C. Sablowski R. (2005). Transcriptional program controlled by the floral homeotic gene AGAMOUS during early organogenesis. *Development* 132 429–438. 10.1242/dev.01600 15634696

[B16] Gross-Hardt R. Lenhard M. Laux T. (2002). WUSCHEL signaling functions in interregional communication during *Arabidopsis* ovule development. *Genes Dev.* 16 1129–1138. 10.1101/gad.225202 12000795 PMC186242

[B17] Huff J. (2015). The Airyscan detector from ZEISS: confocal imaging with improved signal-to-noise ratio and super-resolution. *Nat. Methods* 12 i–ii.

[B18] Ito Y. Nakanomyo I. Motose H. Iwamoto K. Sawa S. Dohmae N. (2006). Dodeca-CLE peptides as suppressors of plant stem cell differentiation. *Science* 313 842–845. 10.1126/science.1128436 16902140

[B19] Kayes J. M. Clark S. E. (1998). CLAVATA2, a regulator of meristem and organ development in *Arabidopsis*. *Development* 125 3843–3851. 10.1242/dev.125.19.3843 9729492

[B20] Kitagawa M. Jackson D. (2019). Control of meristem size. *Annu Rev Plant Biol* 70 269–291. 10.1146/annurev-arplant-042817-040549 31035828

[B21] Lenhard M. Laux T. (2003). Stem cell homeostasis in the *Arabidopsis* shoot meristem is regulated by intercellular movement of CLAVATA3 and its sequestration by CLAVATA1. *Development* 130 3163–3173. 10.1242/dev.00525 12783788

[B22] Liu X. Kim Y. J. Muller R. Yumul R. E. Liu C. Pan Y. (2011). AGAMOUS terminates floral stem cell maintenance in *Arabidopsis* by directly repressing WUSCHEL through recruitment of Polycomb Group proteins. *Plant Cell* 23 3654–3670. 10.1105/tpc.111.091538 22028461 PMC3229141

[B23] Mayer K. F. Schoof H. Haecker A. Lenhard M. Jurgens G. Laux T. (1998). Role of WUSCHEL in regulating stem cell fate in the *Arabidopsis* shoot meristem. *Cell* 95 805–815. 10.1016/s0092-8674(00)81703-1 9865698

[B24] Molnar A. Melnyk C. W. Bassett A. Hardcastle T. J. Dunn R. Baulcombe D. C. (2010). Small silencing RNAs in plants are mobile and direct epigenetic modification in recipient cells. *Science* 328 872–875. 10.1126/science.1187959 20413459

[B25] Moore I. Galweiler L. Grosskopf D. Schell J. Palme K. (1998). A transcription activation system for regulated gene expression in transgenic plants. *Proc. Natl. Acad. Sci. U.S.A.* 95 376–381. 10.1073/pnas.95.1.376 9419383 PMC18229

[B26] Muller R. Bleckmann A. Simon R. (2008). The receptor kinase CORYNE of *Arabidopsis* transmits the stem cell-limiting signal CLAVATA3 independently of CLAVATA1. *Plant Cell* 20 934–946. 10.1105/tpc.107.057547 18381924 PMC2390746

[B27] Ogawa M. Shinohara H. Sakagami Y. Matsubayashi Y. (2008). *Arabidopsis* CLV3 peptide directly binds CLV1 ectodomain. *Science* 319:294. 10.1126/science.1150083 18202283

[B28] ÓMaoiléidigh D. S. Thomson B. Raganelli A. Wuest S. E. Ryan P. T. Kwasniewska K. (2015). Gene network analysis of *Arabidopsis thaliana* flower development through dynamic gene perturbations. *Plant J.* 83 344–358. 25990192 10.1111/tpj.12878

[B29] ÓMaoiléidigh D. S. Wuest S. E. Rae L. Raganelli A. Ryan P. T. Kwasniewska K. (2013). Control of reproductive floral organ identity specification in *Arabidopsis* by the C function regulator AGAMOUS. *Plant Cell* 25 2482–2503. 10.1105/tpc.113.113209 23821642 PMC3753378

[B30] Payne T. Johnson S. D. Koltunow A. M. (2004). KNUCKLES (KNU) encodes a C2H2 zinc-finger protein that regulates development of basal pattern elements of the *Arabidopsis* gynoecium. *Development* 131 3737–3749. 10.1242/dev.01216 15240552

[B31] Prunet N. (2017). Live confocal imaging of the *Arabidopsis* flower. *J. Vis. Exp.* 122:55156. 10.3791/55156 PMC5564460 28448004

[B32] Prunet N. Jack T. P. Meyerowitz E. M. (2016). Live confocal imaging of *Arabidopsis* flower buds. *Dev. Biol.* 419 114–120. 10.1016/j.ydbio.2016.03.018 26992363 PMC5025338

[B33] Prunet N. Yang W. Das P. Meyerowitz E. M. Jack T. P. (2017). SUPERMAN prevents class B gene expression and promotes stem cell termination in the fourth whorl of *Arabidopsis thaliana* flowers. *Proc. Natl. Acad. Sci. U.S.A.* 114 7166–7171. 10.1073/pnas.1705977114 28634297 PMC5502645

[B34] Ryan P. T. ÓMaoiléidigh D. S. Drost H.-G. Kwasniewska K. Gabel A. Grosse I. (2015). Patterns of gene expression during *Arabidopsis* flower development from the time of initiation to maturation. *BMC Genomics* 16:488. 26126740 10.1186/s12864-015-1699-6 PMC4488132

[B35] Schoof H. Lenhard M. Haecker A. Mayer K. F. Jurgens G. Laux T. (2000). The stem cell population of *Arabidopsis* shoot meristems in maintained by a regulatory loop between the CLAVATA and WUSCHEL genes. *Cell* 100 635–644. 10.1016/s0092-8674(00)80700-x 10761929

[B36] Schwab R. Ossowski S. Riester M. Warthmann N. Weigel D. (2006). Highly specific gene silencing by artificial microRNAs in *Arabidopsis*. *Plant Cell* 18 1121–1133. 10.1105/tpc.105.039834 16531494 PMC1456875

[B37] Skene P. J. Henikoff S. (2017). An efficient targeted nuclease strategy for high-resolution mapping of DNA binding sites. *Elife* 6:e21856. 28079019 10.7554/eLife.21856 PMC5310842

[B38] Skopelitis D. S. Hill K. Klesen S. Marco C. F. Von Born P. Chitwood D. H. (2018). Gating of miRNA movement at defined cell-cell interfaces governs their impact as positional signals. *Nat. Commun.* 9:3107. 30082703 10.1038/s41467-018-05571-0 PMC6079027

[B39] Sun B. Looi L. S. Guo S. He Z. Gan E. S. Huang J. (2014). Timing mechanism dependent on cell division is invoked by Polycomb eviction in plant stem cells. *Science* 343:1248559. 10.1126/science.1248559 24482483

[B40] Sun B. Xu Y. Ng K. H. Ito T. (2009). A timing mechanism for stem cell maintenance and differentiation in the *Arabidopsis* floral meristem. *Genes Dev* 23 1791–1804. 10.1101/gad.1800409 19651987 PMC2720260

[B41] Sun B. Zhou Y. Cai J. Shang E. Yamaguchi N. Xiao J. (2019). Integration of transcriptional repression and polycomb-mediated silencing of WUSCHEL in Floral meristems. *Plant Cell* 31 1488–1505. 10.1105/tpc.18.00450 31068455 PMC6635863

[B42] Thomson B. Wellmer F. (2019). Molecular regulation of flower development. *Curr. Top. Dev. Biol.* 131 185–210. 10.1016/bs.ctdb.2018.11.007 30612617

[B43] Triezenberg S. J. Kingsbury R. C. Mcknight S. L. (1988). Functional dissection of VP16, the trans-activator of herpes simplex virus immediate early gene expression. *Genes Dev.* 2 718–729. 10.1101/gad.2.6.718 2843425

[B44] Xu Y. Yamaguchi N. Gan E. S. Ito T. (2019). When to stop: an update on molecular mechanisms of floral meristem termination. *J. Exp. Bot.* 70 1711–1718. 10.1093/jxb/erz048 30916342

[B45] Yadav R. K. Perales M. Gruel J. Girke T. Jonsson H. Reddy G. V. (2011). WUSCHEL protein movement mediates stem cell homeostasis in the *Arabidopsis* shoot apex. *Genes Dev.* 25 2025–2030. 10.1101/gad.17258511 21979915 PMC3197201

[B46] Yamaguchi N. Huang J. Tatsumi Y. Abe M. Sugano S. S. Kojima M. (2018). Chromatin-mediated feed-forward auxin biosynthesis in floral meristem determinacy. *Nat. Commun.* 9:5290. 30538233 10.1038/s41467-018-07763-0 PMC6289996

[B47] Yamaguchi N. Huang J. Xu Y. Tanoi K. Ito T. (2017). Fine-tuning of auxin homeostasis governs the transition from floral stem cell maintenance to gynoecium formation. *Nat. Commun.* 8:1125. 29066759 10.1038/s41467-017-01252-6 PMC5654772

[B48] Yanofsky M. F. Ma H. Bowman J. L. Drews G. N. Feldmann K. A. Meyerowitz E. M. (1990). The protein encoded by the *Arabidopsis* homeotic gene agamous resembles transcription factors. *Nature* 346 35–39. 10.1038/346035a0 1973265

