# Supplementary figures and images for: Expression of KNUCKLES in the Stem Cell Domain Is Required for Its Function in the Control of Floral Meristem Activity in Arabidopsis
Source: Front Plant Sci. 2021 Jul 21;12:704351. doi: 10.3389/fpls.2021.704351 (PMC8336581; doi:10.3389/fpls.2021.704351)

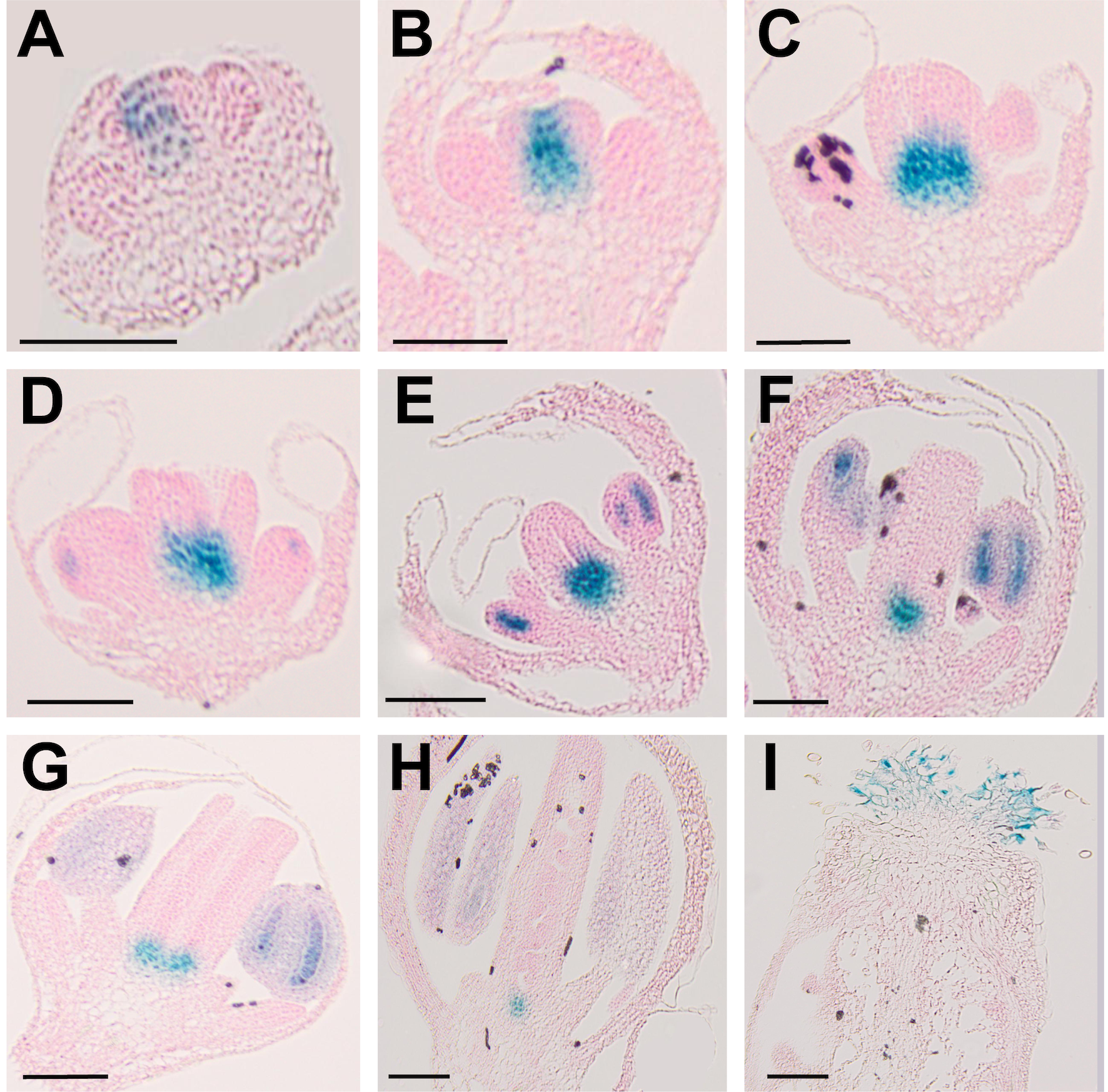

Supplement: Supplementary Figure 1 — Activity of a pKNU::KNU-GUS reporter across flower development. (A,B) GUS activity in the center of a stage 6 (A) and 7 (B) flower. (C–E) Later in development, GUS activity was observed at the base of the gynoecium. (E) At stage 9, GUS activity was also present in the anthers of stamens. (F–I) At later stages (∼10–13), activity of the pKNU::KNU-GUS reporter decreased. (H) GUS activity detected at the base of a developing gynoecium of a stage 11 flower. (I) GUS activity in the stigmatic tissue of mature flowers. Scale bars: 50 μm. [file Image_1.TIF]

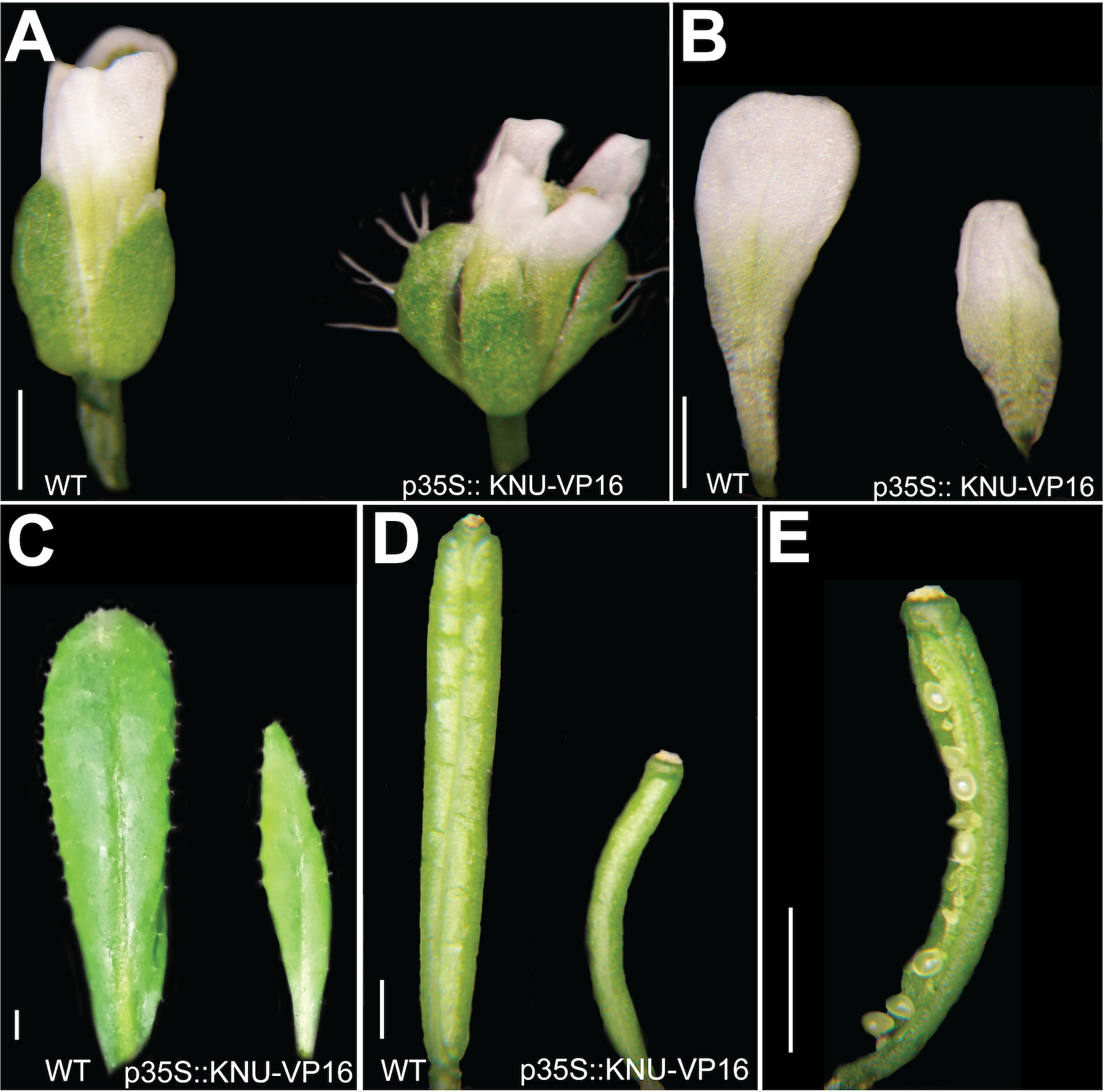

Supplement: Supplementary Figure 2 — Phenotypes of p35S::KNU-VP16 plants. (A) Flower of a transgenic plant with reduced size compared to the wild type, and numerous trichomes on sepals. (B) Size comparison of a wild-type (WT) petal and a petal of a transgenic plant. (C) Plants carrying the p35S::KNU-VP16 transgene produced smaller leaves than the wild type. (D) Siliques of wild-type and p35S::KNU-VP16 plants. (E) Silique of a transgenic plant with reduced seed set. Part of a valve was removed for better visibility. Scale bars: 1 mm. [file Image_2.TIF]

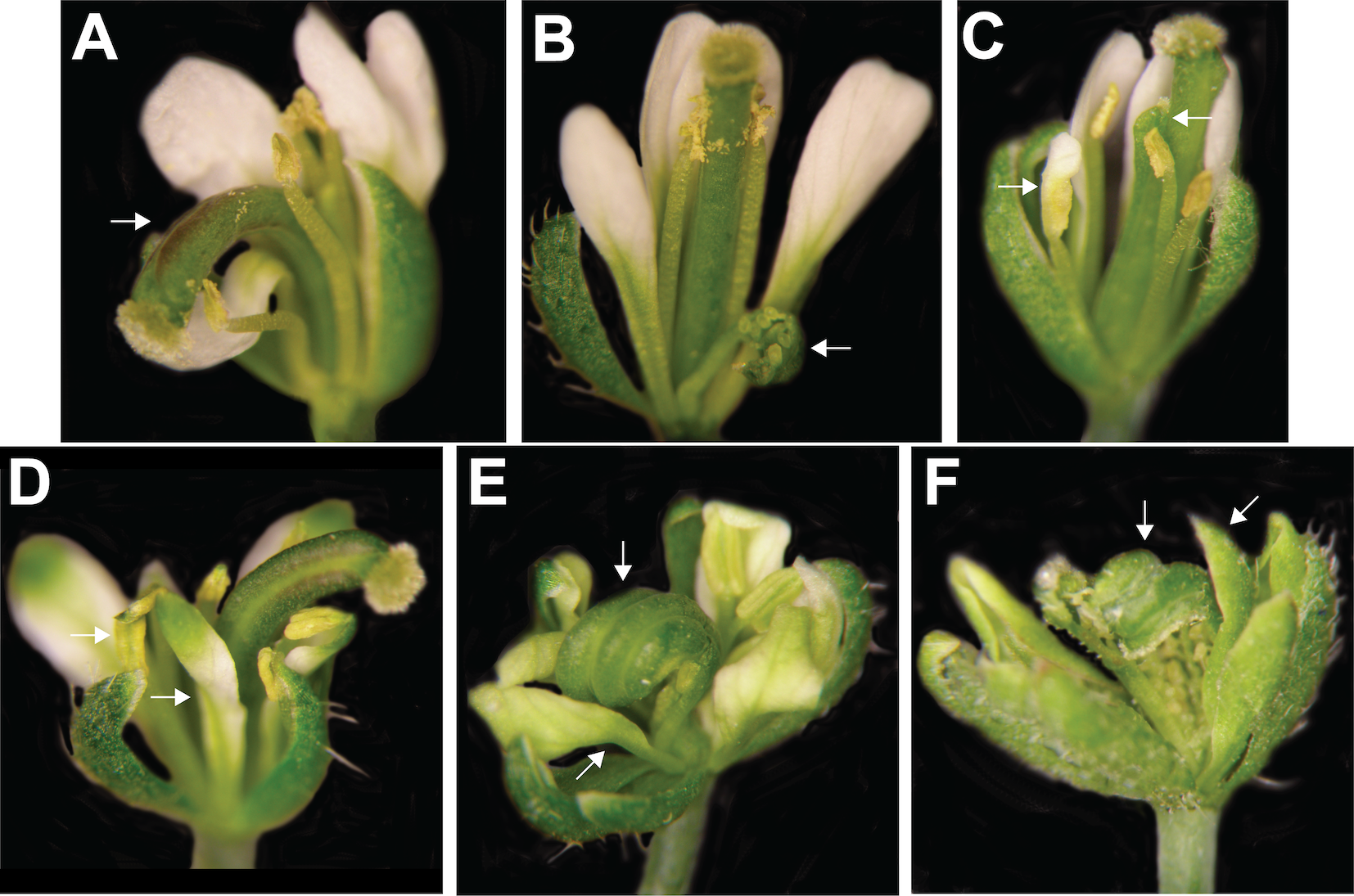

Supplement: Supplementary Figure 3 — Floral phenotypes of p35S::KNU(mEAR)-VP16 plants. (A) Flower of a phenotypically weak line with a bent gynoecium. (B–D) Flowers of lines with intermediate phenotypes with mild homeotic transformations. Arrows indicate a stamen with carpelloid tissue (B); a petalloid stamen (C); and a sepalloid petal (D). (E) Flower from a phenotypically strong plant with misshapen and green petals. The tricarpelloid gynoecium exhibited reduced seed set. (F) Flower with sepal-like organs in place of petals and stamens and an unfused gynoecium. Sepals were removed from all flowers for better visibility of the inner-whorl organs. [file Image_3.TIF]

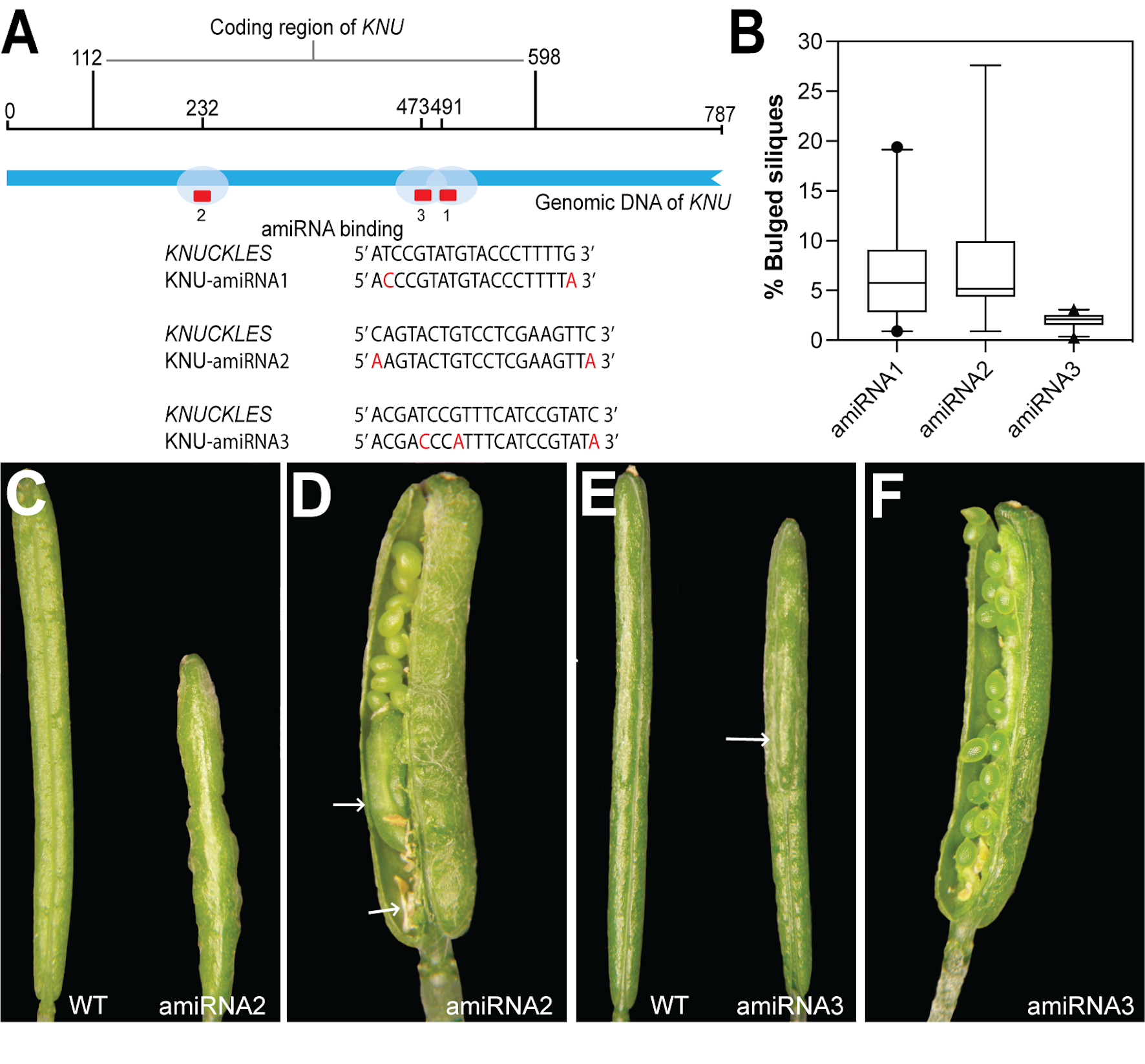

Supplement: Supplementary Figure 4 — Identification of functional KNU-amiRNAs. (A) Schematic drawing showing the sequences of the three amiRNAs tested in this study and the regions in KNU they are targeting. (B) Percentage of bulged siliques formed by lines expressing the different amiRNAs (as indicated). At least 20 independent transformants were analyzed per construct. (C,D) Siliques of p35S::KNU-amiRNA2 plants were bulged and often contained ectopic carpels and stamens (indicated by arrows). (E,F) Siliques of p35S::KNU-amiRNA3 plants. No ectopic organ formation was observed (F), however, siliques were often tricarpelloid (arrow) (E). [file Image_4.TIF]

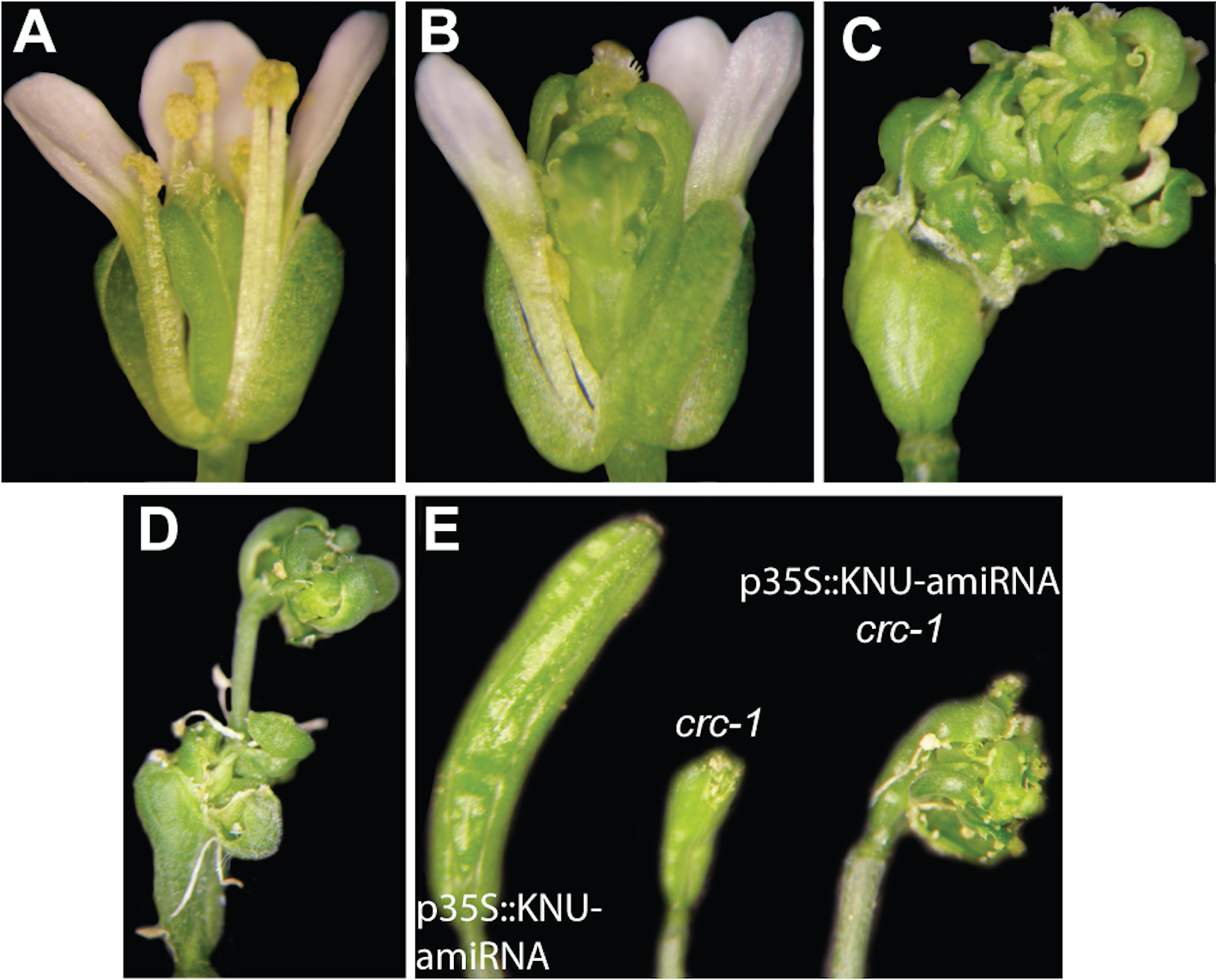

Supplement: Supplementary Figure 5 — Genetic interaction between CRC and KNU in the control of floral meristem activity. (A) Crc-1 mutant flower with unfused carpel at the top of the gynoecium. (B) knu-1 crc-1 double-mutant flower with shorter stamens, which do not produce pollen, and ectopic tissue inside the gynoecium. (C) Early arising silique of the double mutant with an unfused gynoecium and ectopic carpel-like tissue. (D) Silique from a late arising flower producing a new flower from a silique. (E) Siliques of p35S::KNU-amiRNA1 crc-1 plants were similar to siliques of knu-1 crc-1 double mutants - compare to (C). [file Image_5.TIF]

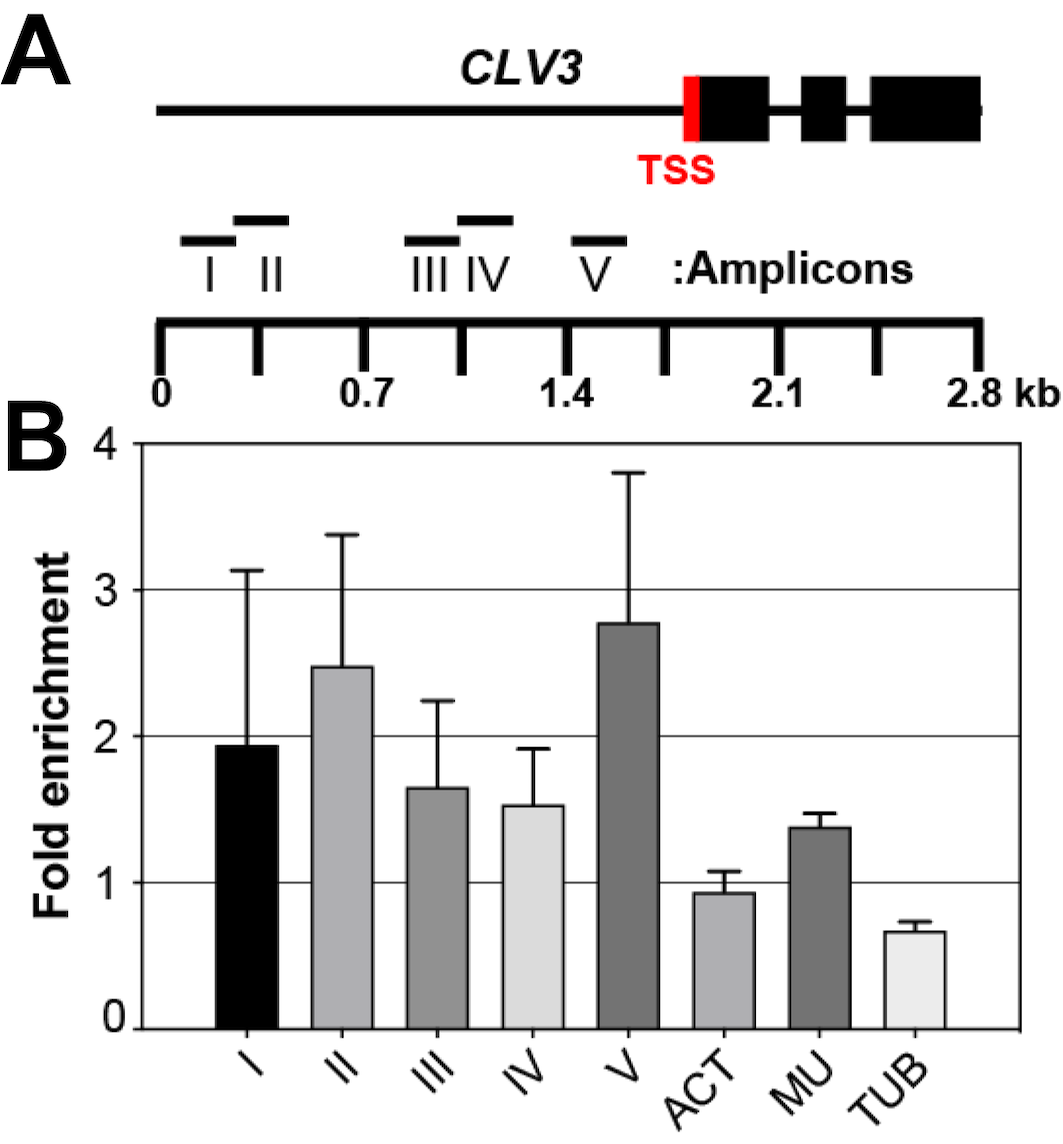

Supplement: Supplementary Figure 6 — Results of chromatin immunoprecipitation experiments. ChIP-qPCR analysis of KNU-GFP binding to the CLV3 promoter region in ∼stage 7-8 floral buds.(A) Position of primer pairs used to analyze KNU-GFP binding across the CLV3 promoter region. (B) ChIP analysis of KNU-GFP binding to the CLV3 promoter locus. Data is shown as fold enrichment of target loci over enrichment of reference genes ACT, MU and TUB (see section “Materials and Methods”). Three independent experiments were performed and the mean value is plotted above with error bars denoting SEM. [file Image_6.TIF]

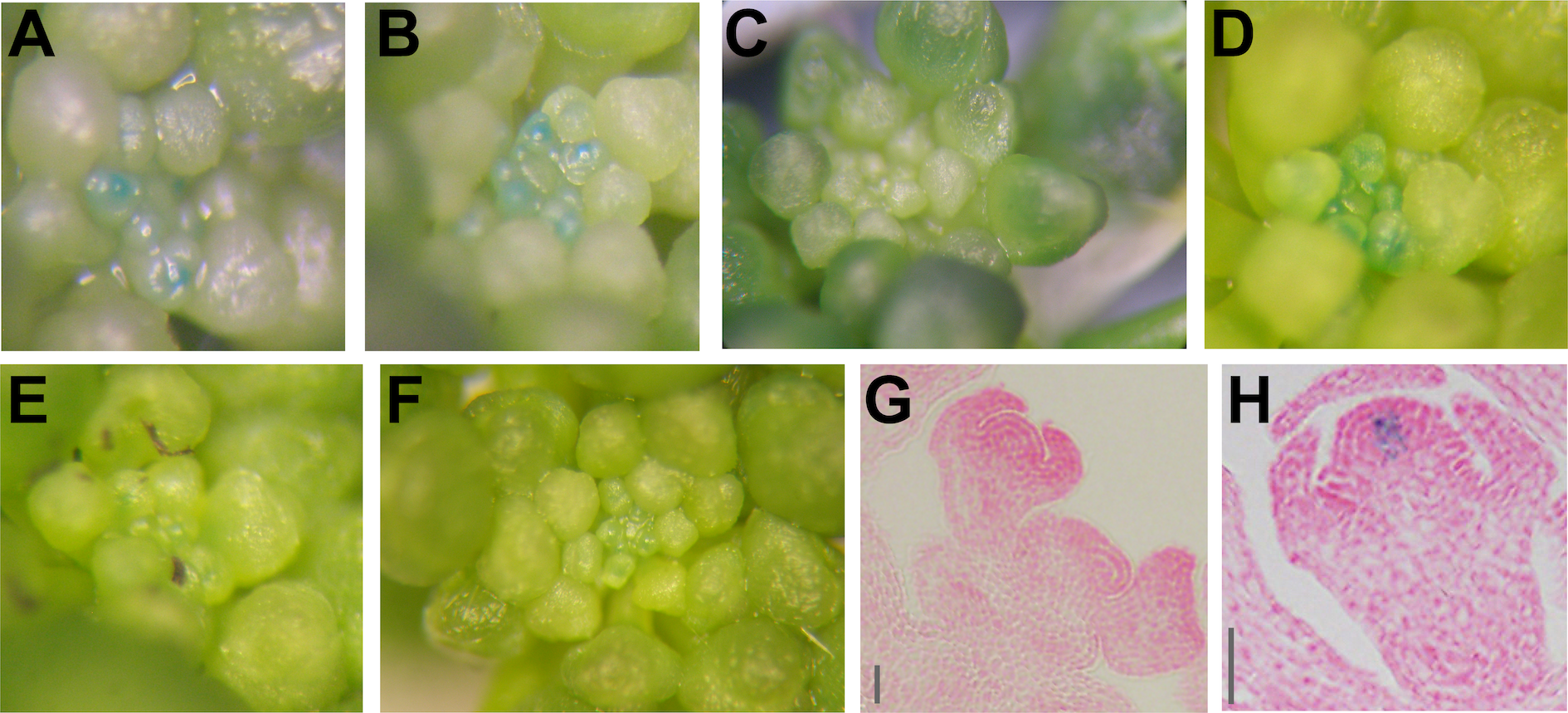

Supplement: Supplementary Figure 7 — Characterization of the strength of the driver lines used. (A,B) Inflorescences of a pWUS>>GUS line after staining for 1 (A) or 2 (B) hours. (C,D) Inflorescences of a pCLV3>>GUS line after staining for 1 (C) or 2 (D) hours. (E,F) Inflorescences of a pCLV1>>GUS line after staining for 1 (E) or 2 (F) hours. (G,H) Inflorescences of a pCLV1>>GUS line were sectioned after staining for 1 (G) or 2 (H) hours. Scale bars: 20 μm. [file Image_7.TIF]
